# Absorption and Emission Spectroscopic Investigation of Thermal Dynamics and Photo-Dynamics of the Rhodopsin Domain of the Rhodopsin-Guanylyl Cyclase from the Nematophagous Fungus *Catenaria anguillulae*

**DOI:** 10.3390/ijms18102099

**Published:** 2017-10-05

**Authors:** Alfons Penzkofer, Ulrike Scheib, Katja Stehfest, Peter Hegemann

**Affiliations:** 1 Fakultät für Physik, Universität Regensburg, Universitätsstraße 31, D-93053 Regensburg, Germany; 2 Experimentelle Biophysik, Institut für Biologie, Humboldt Universität zu Berlin, Invalidenstraße 42, D-10115 Berlin, Germany; ulrike.scheib@hu-berlin.de (U.S.); katja.stehfest@cms.hu-berlin.de (K.S.); hegemann@rz.hu-berlin.de (P.H.)

**Keywords:** rhodopsin domain, rhodopsin-guanylyl cyclase, nematophagous fungus *Catenaria anguillulae*, retinal Schiff base, primary photocycle dynamics, secondary photocycle dynamics, photo-degradation

## Abstract

The rhodopsin-guanylyl cyclase from the nematophagous fungus *Catenaria anguillulae* belongs to a recently discovered class of enzymerhodopsins and may find application as a tool in optogenetics. Here the rhodopsin domain CaRh of the rhodopsin-guanylyl cyclase from *Catenaria anguillulae* was studied by absorption and emission spectroscopic methods. The absorption cross-section spectrum and excitation wavelength dependent fluorescence quantum distributions of CaRh samples were determined (first absorption band in the green spectral region). The thermal stability of CaRh was studied by long-time attenuation measurements at room temperature (20.5 °C) and refrigerator temperature of 3.5 °C. The apparent melting temperature of CaRh was determined by stepwise sample heating up and cooling down (obtained apparent melting temperature: 62 ± 2 °C). The photocycle dynamics of CaRh was investigated by sample excitation to the first inhomogeneous absorption band of the CaRh_da_ dark-adapted state around 590 nm (long-wavelength tail), 530 nm (central region) and 470 nm (short-wavelength tail) and following the absorption spectra development during exposure and after exposure (time resolution 0.0125 s). The original protonated retinal Schiff base PRSB_all*-trans*_ in CaRh_da_ photo-converted reversibly to protonated retinal Schiff base PRSB_all*-trans*,la1_ with restructured surroundings (CaRh_la1_ light-adapted state, slightly blue-shifted and broadened first absorption band, recovery to CaRh_da_ with time constant of 0.8 s) and deprotonated retinal Schiff base RSB_13*-cis*_ (CaRh_la2_ light-adapted state, first absorption band in violet to near ultraviolet spectral region, recovery to CaRh_da_ with time constant of 0.35 s). Long-time light exposure of light-adapted CaRh_la1_ around 590, 530 and 470 nm caused low-efficient irreversible degradation to photoproducts CaRh_prod_. Schemes of the primary photocycle dynamics of CaRh_da_ and the secondary photocycle dynamics of CaRh_la1_ are developed.

## 1. Introduction

The nematophagous fungus *Catenaria anguillulae* is a facultative endoparasite of free living and plant parasitic nematodes ([1,2] and references therein). Searches of the genome assembly of *Catenaria anguillulae* found the presence of a rhodopsin-guanylyl cyclase gene fusion [3]. This guanylyl cyclase opsin, named CaCyclOp in [4], was expressed and preliminarily characterized respective cGMP (cyclic guanosine monophosphate) production in *Xenopus* oocytes [4]. A more extensive study of rhodopsin-cyclases for light-induced cGMP and cAMP (cyclic adenosine monophosphate) production was carried out with the related rhodopsin-guanylyl cyclase from *Blastocladiella emersonii* BeRhGC [4,5]. However recombinant full-length BeRhGC was thermally unstable and light-induced cyclase activity could not be measured. However, the recombinant rhodopsin fragment BeRh was characterized respective its photophysical and photochemical properties [5,6]. Moreover, the full-length protein BeRhGC could be functionally expressed in hippocampal neurons, making it usable to optogenetic applications [5].

Here absorption and fluorescence spectroscopic characterizations of the rhodopsin domain CaRh of the rhodopsin-guanylyl cyclase CaRhGC from *Catenaria anguillulae* were performed. In all experiments the CaRh protein was dissolved in pH 7.3 HEPES/MOPS buffer with DDM and CHS detergents (HEPES = 4-(2-hydroxyethyl)-1-piperazineethanesulfonic acid, MOPS = 3-(*N*-morpholino) propanesulfonic acid, DDM = n-dodecyl β-d-maltoside, CHS = cholesteryl hemisuccinate, for exact composition see below Materials and Methods). The amino acid sequence of CaRh is displayed in Figure S1 and is described in Supplementary Material S1. The thermal stability, the photocycle dynamics, and the photo-degradation of CaRh were studied by absorption spectroscopic methods. The time resolution of the photocycle investigations was 0.0125 s. A high thermal stability of CaRh was observed with apparent protein melting temperature of ϑ_m_ = 62 ± 2 °C. In the dark-adapted state (dark-adapted state CaRh_da_) the covalently bound protonated retinal Schiff base cofactor PRSB_all*-trans*_ has a strongly inhomogeneous broadened singlet S_0_–S_1_ absorption band indicating a diverse conformational protein structure distribution and charge distribution modifying the retinal absorption wavelength positions. In the photocycle studies photo-excitation caused reversible excited-state PRSB_all*-trans*_ → PRSB_13*-cis*_ all*-trans*—13*-cis* isomerization with subsequent deprotonation to RSB_13*-cis*_ (CaRh_la2_ light-adapted state formation) and all*-trans* back-isomerization with protein conformation and charge distribution changes blue-shifting and spectral broadening the S_0_–S_1_ absorption band shape (CaRh_la1_ light-adapted state formation). Photo-excitation of CaRh_la1_ did not cause further reversible CaRh_la2_ formation. Long-time intense photo-excitation (CaRh in CaRh_la1_ state) caused low-efficient irreversible formation of photoproducts CaRh_Prod_.

For convenience full names of abbreviations, indices, and symbols are collected at the end of the paper.

## 2. Results

### 2.1. Absorption and Emission Behavior of Freshly Thawed CaRh

#### 2.1.1. Absorption Behavior of CaRh

**Figure 1:**
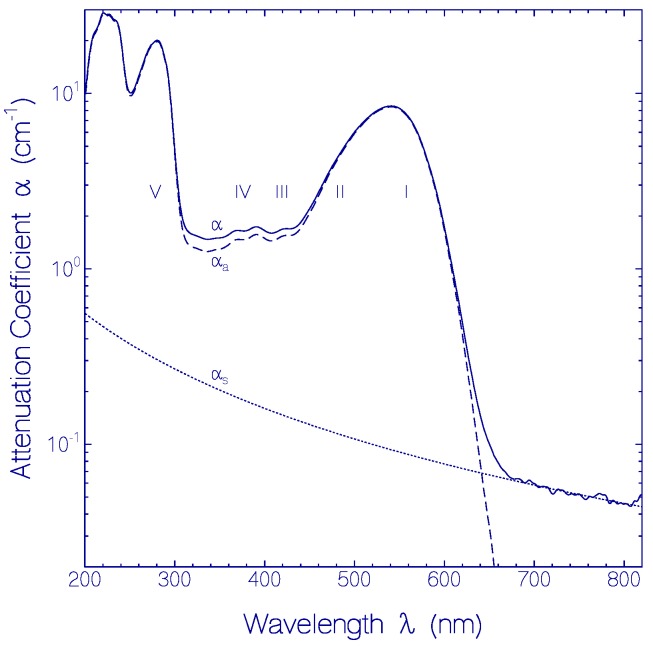
Attenuation coefficient spectrum of a fresh CaRh sample in pH 7.3 HEPES/MOPS buffer. Solid curve: attenuation coefficient spectrum α(*λ*) measured after sample centrifugation (4400 rpm for 15 min at 4 °C). Dotted curve: approximate scattering contribution α_s_(*λ*). Dashed curve: approximate absorption coefficient spectrum α_a_(*λ*). The numbers I–V indicate different excitation transitions.

The attenuation coefficient spectrum α(*λ*) of a fresh CaRh sample measured after thawing and centrifugation at 4400 rpm for 15 min at 4 °C is displayed by the solid curve in Figure 1. The dotted curve shows the light scattering contribution α_s_(*λ*) fitted by [7] $\alpha_{s}(\lambda )=\alpha (\lambda_{0}){\left(\lambda_{0}/\lambda \right)}^{\gamma }$ with λ_0_ = 800 nm, α(λ_0_) = 0.046 cm^−1^ and γ = 1.8. The dashed curve shows the absorption coefficient contribution α_a_(λ) = α(λ) − α_s_(λ) of CaRh. The absorption coefficient spectrum of CaRh is composed of the retinal absorption in the wavelength region of λ > 330 nm, and the combined apoprotein and retinal absorption in the wavelength region of λ < 330 nm. The absorption cross-section spectrum of CaRh is determined in Supplementary Material S2 and displayed in the upper part of Figure S2 (solid curve). The light scattering situation is discussed in Supplementary Material S3.

The retinal absorption coefficient spectrum of CaRh reveals an inhomogeneous broadened S_0_–S_1_ absorption band with peak position at *λ*_S0–S1,peak_ = 541 nm and spectral wavenumber half-width of $\Delta {\tilde{\nu }}_{S_{0}-S_{1}}$ ≈ 3600 cm^−1^ (FWHM). The absorption band localization in the green spectral region indicates the presence of the retinal cofactor as protonated retinal Schiff base PRSB [8]. The inhomogeneous broadening indicates different embedding of protonated retinal Schiff base in the rhodopsin apoprotein environment with varying amino acid charge distribution and possibly different retinal conformational structures (isomers). The short-wavelength absorption peaks at *λ* = 424 and 391 nm are thought to be peak absorption wavelength positions of less inhomogeneous broadened S_0_–S_2_ and the S_0_–S_3_ transitions of PRSB. The absorption modulation around 370, 348 and 322 nm may indicate the vibronic structure of the S_0_–S_3_ absorption band.

#### 2.1.2. Fluorescence Behavior of CaRh

**Figure 2:**
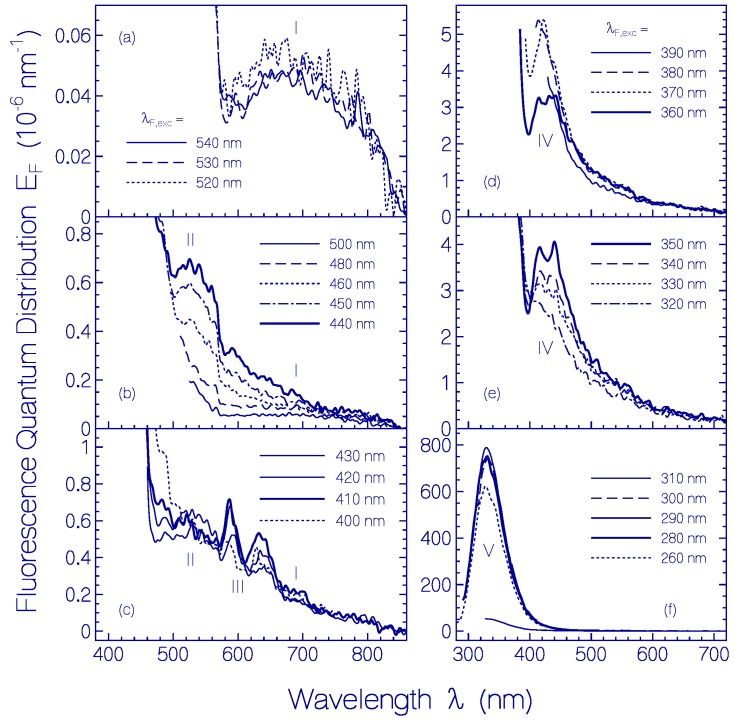
Fluorescence emission quantum distributions *E*_F_(*λ*) of fresh centrifuged CaRh in pH 7.3 HEPES/MOPS buffer for various fluorescence excitation wavelengths in the regions from (**a**) 540 to 520 nm; (**b**) 500 to 440 nm; (**c**) 430 to 400 nm; (**d**) 390 to 360 nm; (**e**) 350 to 320 nm, and (**f**) 310 to 260 nm. Numbers I–V indicate origins from different excitation transitions.

**Figure 3:**
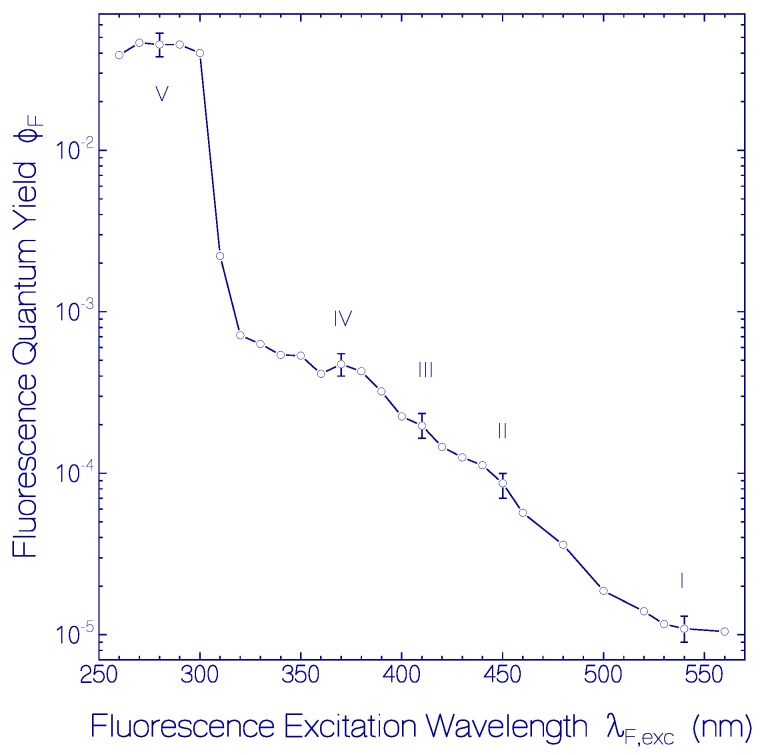
Dependence of fluorescence quantum yield Φ_F_ of fresh CaRh in pH 7.3 HEPES/MOPS buffer versus fluorescence excitation wavelength *λ*_F,exc_ in the region from 560 to 260 nm. Numbers I–V indicate origins from different excitation transitions.

Fluorescence emission quantum distributions *E*_F_(*λ*) of the fresh centrifuged CaRh sample used in Figure 1 at excitation wavelengths in the region from *λ*_F,exc_ = 540 to 260 nm are displayed in Figure 2 with sub-sets (a)–(f). The corresponding fluorescence quantum yield curve in the range from *λ*_F,exc_ = 560 to 260 nm is shown in Figure 3.

Over the broad inhomogeneous S_0_–S_1_ absorption band the fluorescence quantum distributions change their spectral shapes and magnitudes. In the long-wavelength excitation region *λ*_F,exc_ > 520 nm of the S_0_–S_1_ absorption band (Figure 2a), a well-shaped single fluorescence emission band is resolved with fluorescence maximum around 690 nm (fluorescence Stokes shift $\delta {\tilde{\nu }}_{St}=\lambda_{a,\max}^{-1}-\lambda_{F,\max}^{-1}$ ≈ 4000 cm^−1^, spectral width $\Delta {\tilde{\nu }}_{F}$ ≈ 6000 cm^−1^ (FWHM)). In the short-wavelength region 440 nm < *λ*_F,exc_ < 500 nm of the S_0_–S_1_ absorption band (Figure 2b) a second fluorescence emission band with up to a factor of ten higher magnitude and smaller spectral width is present (*λ*_F,max_ ≈ 525 nm, $\Delta {\tilde{\nu }}_{F}$ ≈ 3000 cm^−1^). In the case of fluorescence excitation in the range from 430 to 400 nm (Figure 2c, dominant excitation of the S_0_–S_2_ absorption band of PRSB) the fluorescence spectra include a well resolved vibronic structure with peaks at ≈588 and ≈637 nm (vibronic wavenumber spacing ≈ 1300 cm^−1^). Fluorescence excitation in the wavelength range from 390 to 320 nm (Figure 2d,e) seems to be dominated by PRSB S_3_–S_0_ emission with peak fluorescence emission wavelength of *λ*_F,max_ ≈ 425 nm and full spectral half-width of $\Delta {\tilde{\nu }}_{F}$ ≈ 4300 cm^−1^. For fluorescence excitation in the range from 310 to 260 nm (Figure 2f) the fluorescence is dominated by Trp (tryptophan) emission of the rhodopsin apoprotein peaking at *λ*_F,max_ ≈ 330 nm (full spectral half-width $\Delta {\tilde{\nu }}_{F}$ ≈ 5000 cm^−1^). The Tyr (tyrosine) emission (expected fluorescence peak emission around 308 nm) is quenched by Förster-type energy transfer from Tyr to Trp (see supplementary material to [9]).

The fluorescence quantum yield of CaRh peaking around *λ*_F,max_ ≈ 690 nm (*λ*_F,exc_ around 540 nm) is Φ_F,I_ = Φ_F_ (PRSB, S_1_–S_0_, long-wavelength part) = (1.1 ± 0.2) × 10^−5^. The fluorescence quantum yield of CaRh peaking around *λ*_F,max_ ≈ 525 nm (*λ*_F,exc_ around 450 nm) is Φ_F,II_ = Φ_F_ (PRSB, S_1_–S_0_, short-wavelength part) = (8.5 ± 0.15) × 10^−5^. The fluorescence quantum yield in the case of excitation around 410 nm is Φ_F,III_ = Φ_F_ (PRSB, S_2_–S_0_) = (2.0 ± 0.35) × 10^−4^. Fluorescence excitation around 370 nm gives Φ_F,IV_ = Φ_F_ (PRSB, S_3_–S_0_) = (4.75 ± 0.75) × 10^−4^. Fluorescence excitation around 280 nm is dominated by apoprotein emission with a fluorescence quantum yield of Φ_F,V_ = Φ_F_ (apoprotein) = 0.045 ± 0.007.

The fluorescence lifetimes (lifetimes of emitting states) of the considered transitions *i* = I–V are determined by the fluorescence quantum yields Φ_F,i_ and the radiative lifetimes *τ*_rad,i_ according to

(1) $$\tau_{F,i}=\Phi_{F,i}\tau_{rad,i}$$

The radiative lifetimes of the transitions are given by the Strickler-Berg relation [10,11,12]

(2) $$\tau_{rad,i}=\frac{n_{a,i}{\bar{\lambda }}_{F,i}^{3}}{8\pi c_{0}n_{F,i}^{3}{\bar{\sigma }}_{a,i}}$$

where *n*_a.i_ and *n*_F,i_ are the mean refractive indices in the absorption band region and the fluorescence region of the considered transitions, respectively (determined by the water solvent), ${\bar{\lambda }}_{F,i}={\left[\int E_{F,i}(\lambda )\lambda^{3}d\lambda /\int E_{F,i}(\lambda )d\lambda \right]}^{1/3}$ are the mean fluorescence wavelengths, and ${\bar{\sigma }}_{a,i}=\int_{}\left[\sigma_{a,i}(\lambda )/\lambda \right]d\lambda$ are the absorption band cross-section strengths of the considered transitions.

In Table 1 approximate and estimated values of absorption wavelength positions *λ*_a,i_, peak fluorescence emission wavelengths *λ*_F,max,i_, fluorescence spectral half-widths $\Delta {\tilde{\nu }}_{F,i}$ (FWHM), mean fluorescence wavelengths ${\bar{\lambda }}_{F,i}$, mean refractive indices *n*_a,i_ and *n*_F,i_, fluorescence quantum yields Φ_F,i_, absorption band cross-section strengths of considered bands ${\bar{\sigma }}_{a,i}$, radiative lifetimes *τ*_rad,i_, and Strickler-Berg based fluorescence lifetimes *τ*_F,i_ of the transitions *i* = I–V are collected. The S_0_–S_1_ absorption band cross-section strength ${\bar{\sigma }}_{a}$ of the retinal species PRSB is ${\bar{\sigma }}_{a}={\bar{\sigma }}_{a,I}={\bar{\sigma }}_{a,II}$ = (3.3 ± 0.3) × 10^−17^ cm^2^. It was determined from Figure S2 using ${\bar{\sigma }}_{a}=\int_{\lambda \geq 440\;\mathrm{nm}}\left[\sigma_{a}(\lambda )/\lambda \right]d\lambda$. The S_0_–S_2_ and S_0_–S_3_ absorption band cross-section strengths ${\bar{\sigma }}_{a,III}$ and ${\bar{\sigma }}_{a,IV}$ are thought to be roughly a factor of ten smaller than the S_0_–S_1_ absorption band cross-section strength considering the smaller absorption cross-section peak heights and the smaller inhomogeneous broadening. The value of ${\bar{\sigma }}_{a,V}$ was determined using the absorption cross-section spectrum of Trp, i.e., ${\bar{\sigma }}_{V}={\bar{\sigma }}_{\mathrm{Trp}}=\int_{\lambda \geq 240\;\mathrm{nm}}\left[\sigma_{\mathrm{Trp}}(\lambda )/\lambda \right]d\lambda$ (from [13], incoherent independent emission of each Trp residue in the protein).

The fluorescence lifetime *τ*_F,I_ of the long-wavelength part of the inhomogeneous S_0_–S_1_ transition is found to be *τ*_F,I_ ≈ 88 fs. The fluorescence lifetime *τ*_F,II_ of the short-wavelength part of the inhomogeneous S_0_–S_1_ transition is determined to be *τ*_F,II_ ≈ 305 fs. These short fluorescence lifetimes agree with barrier-less first excited state twist to funnel positions (conical intersection [14]) where fast internal conversion from the excited state S_1_ potential energy surface to the S_0_ ground-state potential energy surface occurs (twisted internal conversion [15]) with partial transfer to products (photo-isomers) and partial recovery to the initial educt conformations [15]. The inhomogeneous S_0_–S_1_ absorption band and different short-wavelength and long-wavelength excitation S_1_–S_0_ fluorescence emission bands indicate different retinal relaxation dynamics caused by the surrounding inhomogeneous opsin protein arrangement.

The higher excited-state S_2_–S_0_ (III) (*τ*_F,III_ ≈ 7.4 ps) and S_3_–S_0_ (IV) (*τ*_F,IV_ ≈ 13.7 ps) picosecond fluorescence lifetimes indicate activation-barrier-slowed-down S_2_ and S_3_ potential energy surface relaxation twists to conical intersections with internal conversion to the S_0_ potential energy surface.

The apoprotein (V) fluorescence behavior is determined by Trp emission (fluorescence quantum yield Φ_F,V_ ≈ 0.045, fluorescence lifetime *τ*_F,V_ ≈ 448 ps). The fluorescence efficiency is reduced and the fluorescence lifetime is shortened by Förster-type energy transfer from apoprotein to retinal [16,17]. The fluorescence quantum yield of Trp outside the protein in aqueous solution is Φ_F_ = 0.13 [18,19,20].

### 2.2. Thermal CaRh Behavior

The CaRh apparent melting temperature and the temporal attenuation behavior at the fixed temperatures of 3.5 and 20.5 °C were studied.

#### 2.2.1. Apparent CaRh Melting Temperature

The thermal protein stability of CaRh was studied by stepwise sample heating up to 69.2 °C, then cooling down and thereby measuring the attenuation coefficient spectra development [6,21]. The apparent protein melting temperature was derived from the onset of strong light attenuation in the transparency region of CaRh with inverse wavelength power dependence of α_s_(*λ*) ∝ *λ*^−*γ*^ (*γ* ≤ 4) [7].

**Figure 4:**
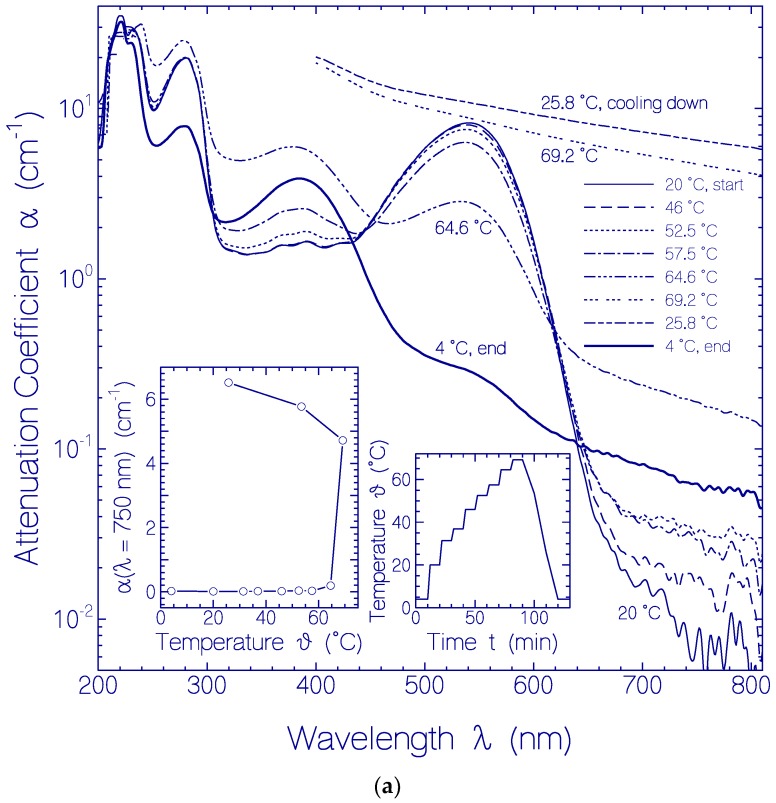

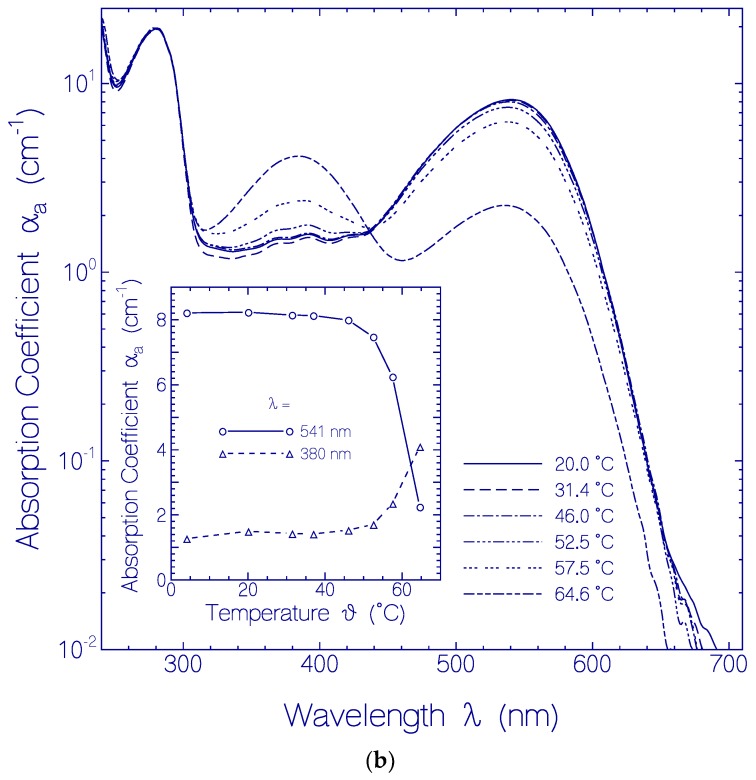
(**a**) Heating-cooling cycle behavior of a fresh thawed CaRh sample in pH 7.3 HEPES/MOPS buffer. Main figure: Attenuation coefficient spectra α(*λ*) development during stepwise sample heating up and cooling down. Right inset: Applied heating and cooling temperature profile *ϑ*(*t*). Left inset: Temperature dependent attenuation coefficient α(*λ* = 750 nm) development; (**b**) Absorption coefficient development of CaRh in pH 7.3 HEPES/MOPS buffer during sample heating up (data from Figure 4a deprived from scattering contribution). Inset: Temperature dependent absorption coefficient development at *λ* = 541 and 380 nm.

The situation is displayed in Figure 4a. The applied heating-cooling cycle is shown by the right inset in Figure 4a. The main part of Figure 4a shows attenuation coefficient spectra of CaRh at fixed temperatures during sample heating up and cooling down. In the transparency region (*λ* > 640 nm) the attenuation coefficient (light scattering) increased during heating up. Strong onset of light scattering was observed for ϑ = 64.6 °C. Between ϑ = 64.6 °C and *ϑ* = 69.2 °C the light attenuation at *λ* = 700 nm increased more than a factor of 20 within a time interval of 10 min. The increase in light scattering is due to protein unfolding and concurring aggregation [21]. The attenuation continued to increase during sample cooling, showing that the protein heat denaturation was irreversible. Only due to sample centrifugation at *ϑ* = 4 °C (4400 rpm for 30 min), the light attenuation was strongly reduced due to sedimentation of aggregated CaRh.

The attenuation coefficient development at *λ* = 750 nm versus temperature during heating and cooling is shown in the left inset of Figure 4a. The temperature position of steep onset of light attenuation gives the apparent protein melting temperature *ϑ*_m_. A value of *ϑ*_m_ = 62 ± 2 °C is determined. At *ϑ*_m_ the protein begins to unfold (denature) quickly with time which causes a quick light-scattering increase with time (the expression “protein melting” is synonymously used to the expression “protein denaturation” [22]).

Concurrent with the sample heating CaRh retinal conversion occurred from protonated retinal Schiff base (PRSB) with peak absorption at *λ*_max_ = 541 nm to unprotonated retinal Schiff base (RSB) with peak absorption at *λ*_max_ = 384 nm. This change is seen by looking to the attenuation coefficient spectra in the main part of Figure 4a. It is more clearly worked out in the main part of Figure 4b where the absorption coefficient spectra development deprived from the scattering contributions is displayed. The inset in Figure 4b shows the development of α_a_(541 nm) and α_a_(380 nm) versus temperature. Above *ϑ* = 50 °C strong conversion of PRSB to RSB is observed.

The absorption cross-section spectrum of CaRh with the retinal cofactor converted from PRSB to RSB is shown by the dotted curve in the top part of Figure S2 (PRSB contribution at *ϑ* = 64.6 °C is subtracted and the resulting spectrum is normalized to 100% RSB content). The long-wavelength absorption cross-section tail for *λ* > 450 nm is thought to be caused by the presence of some released free protonated retinal Schiff base [23].

The fluorescence quantum distribution of the deprotonated retinal Schiff base RSB of the heat-denatured and centrifuged CaRh sample was determined for fluorescence excitation at *λ*_F,exc_ = 360 nm. The obtained spectrum is shown in Figure S3. The fluorescence emission peaks at ≈ 550 nm. The fluorescence quantum yield is Φ_F_ = (1.5 ± 0.2) × 10^−3^. A radiative lifetime of *τ*_rad_ = 7.17 ns, and a fluorescence lifetime of *τ*_F_ = 10.8 ± 1.5 ps are calculated using Equations (1) and (2) with ${\bar{\lambda }}_{F,CaRh,\;heat-denatured}$ = 615 nm and ${\bar{\sigma }}_{CaRh,\;heat-denatured}$ = 2.44 × 10^−17^ cm^2^.

#### 2.2.2. Temporal Absorption Development of CaRh at 3.5 °C

**Figure 5:**
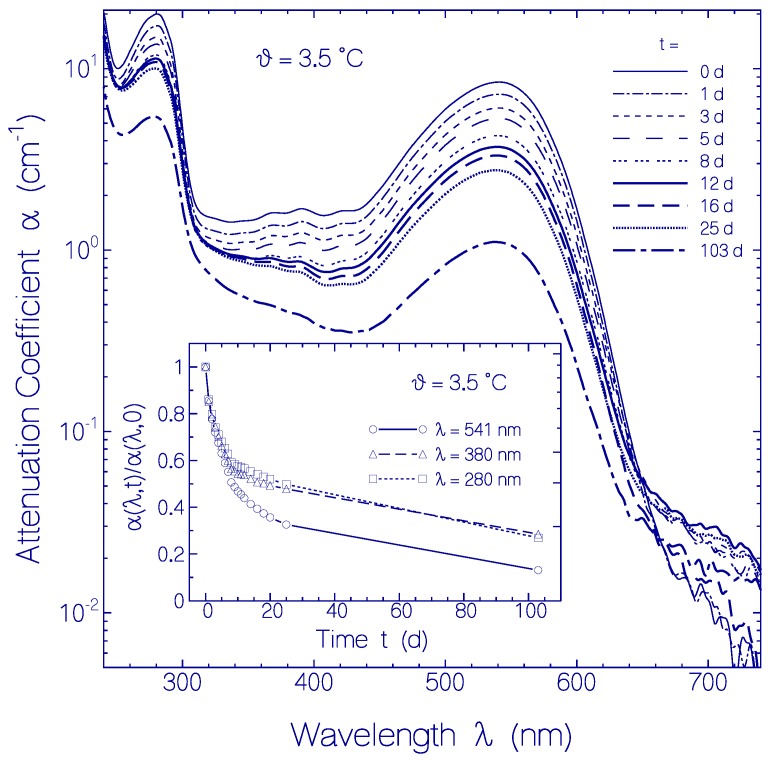
Temporal attenuation coefficient spectra development of CaRh in pH 7.3 HEPES/MOPS buffer at 3.5 °C. Main part: Attenuation coefficient spectra measured after indicated storage times. Inset: Attenuation coefficient ratio α(*λ*,*t*)/α(*λ*,*t* = 0) for the wavelengths *λ* = 541 nm (circles), 380 nm (triangles), and 280 nm (squares).

The attenuation coefficient development α(*λ*) at *ϑ* = 3.5 ± 0.5 °C with time is displayed in Figure 5. The light scattering contribution α_s_(*λ*) to the attenuation coefficient spectra is small as is seen in the long-wavelength transparency region of *λ* > 640 nm. The attenuation coefficient spectra in the absorption region decrease in height with storage time at 3.5 °C. This attenuation reduction is attributed to CaRh aggregate cluster compactization with storage time (loosely packed globules with small volume fill factor densify to tightly packed globules) [24]. The apparent absorption cross-section per molecule decreases because of specific surface reduction of the aggregates (for detailed discussion of aggregation dependent absorption reduction see reference [24]).

The inset in Figure 5 shows the attenuation coefficient development with time for the wavelengths *λ* = 541 nm (PRSB S_0_–S_1_ absorption peak), 380 nm (higher excited-state PRSB absorption and RSB S_0_–S_1_ absorption build-up), and 280 nm (dominant apoprotein Trp and Tyr absorption). The ratios α(*λ*,*t*)/α(*λ*,*t* = 0) are shown. For *t* > 12 days the attenuation ratio decreases more strongly at 541 nm than at 380 nm because of some conversion of PRSB to RSB.

#### 2.2.3. Temporal Absorption Development of CaRh at 20.5 °C

**Figure 6:**
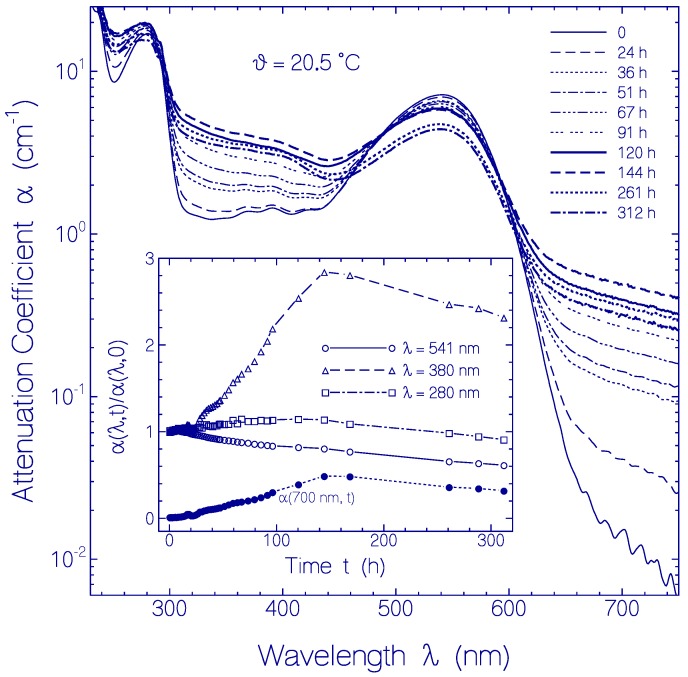
Temporal attenuation coefficient spectra development of CaRh in pH 7.3 HEPES/MOPS buffer at 20.5 °C. Main part: Attenuation coefficient spectra measured after indicated storage times. Inset: Attenuation coefficient ratio α(*λ*,*t*)/α(*λ*,*t* = 0) for the wavelength *λ* = 541 nm (circles), 380 nm (triangles), and 280 nm (squares). The dots show the temporal attenuation coefficient development α(*λ* = 700 nm) in the transparency region of CaRh.

The attenuation coefficient development α(*λ*) at *ϑ* = 20.5 ± 1 °C with time is displayed in Figure 6. In the main part attenuation coefficient spectra at various storage times are shown. At start the light scattering contribution is negligible. Up to *t* = 144 h the light scattering increased as is seen in the transparency region of *λ* > 660 nm. This scattering increase indicates some slow protein denaturation at room temperature. For *t* > 144 h the light attenuation in the transparency region decreased with time likely due to sedimentation of aggregated (denaturated) protein. The attenuation of the main absorption band around 540 nm decreased with time due to protein aggregation (mainly absorption reduction due to protein aggregate cluster compactization [24]) and due to conversion of PRSB to RSB. The attenuation in the region between 300 and 460 nm increases with time during the first 144 h because of increasing light scattering and conversion of PRSB to RSB. For *t* >144 h some attenuation reduction is seen likely due to protein aggregate sedimentation. In the dominant apoprotein absorption region around 280 nm the aggregation dependent absorption coefficient reduction is mainly compensated by aggregation dependent light scattering increase.

In the inset of Figure 6, the temporal attenuation coefficient development at *λ* = 541 nm (S_0_–S_1_ absorption peak of PRSB), *λ* = 380 nm (higher excited-state absorption of PRSB and S_0_–S_1_ absorption of formed RSB), and *λ* = 280 nm (dominant apoprotein Trp and Tyr absorption) is depicted. The ratio α(*λ*,*t*)/α(*λ*,*t* = 0) is plotted. As described above, α(541 nm) decreased with time due to absorption reduction by aggregate compactization, PRSB conversion to RSB, and final aggregate sedimentation. α(380 nm) increased with time during the first 144 h due to dominant light scattering increase over aggregation dependent absorption coefficient reduction and because of PRSB conversion to RSB. For *t* > 144 h the decrease of α(380 nm) is thought to be due to protein aggregate sedimentation. α(280 nm) increased slightly within the first 120 h due to slightly dominating attenuation increase by scattering over absorption reduction by aggregate compactization. For *t* > 144 h the decrease of α(280 nm) is thought to be due to protein aggregate sedimentation. The attenuation coefficient α(700 nm) in the transparency region of CaRh is caused by light scattering. The increase of α(700 nm) during the first 144 h is due to growth of aggregate size. The slight decrease for *t* > 144 h is due to beginning protein aggregate sedimentation.

### 2.3. Photo-Excitation Dynamics of CaRh

**Figure 7:**
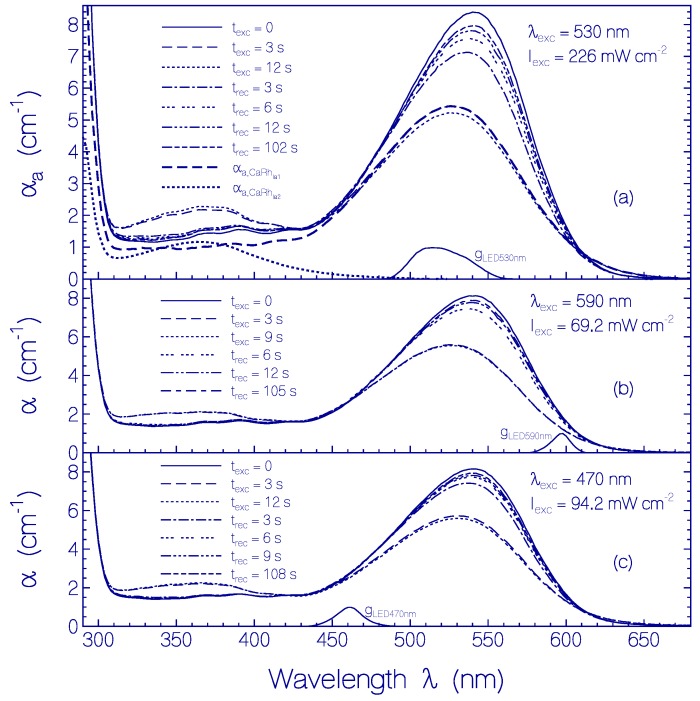
Photocycle behavior of CaRh. Absorption or attenuation coefficient spectra of a CaRh sample in pH 7.3 HEPES/MOPS buffer are shown before excitation (*t*_exc_ = 0 s), during excitation, and after excitation switch-off. (**a**) Excitation with Thorlabs LED 530 nm. Entrance excitation intensity *I*_exc_ = 226 mW cm^−2^. g_LED530nm_(*λ*) = S_LED530nm_(*λ*)/S_LED530nm_(*λ*_max_) is the spectral distribution of the excitation light source. The thick dashed curve shows the separated absorption coefficient contribution $\alpha_{a,CaRh_{\;la1}}$ of CaRh_la1_ at *t*_exc_ = 3 s. The thick dotted curve shows the separated absorption coefficient contribution $\alpha_{a,CaRh_{\;la2}}$ of CaRh_la2_ at *t*_exc_ = 3 s; (**b**) Excitation with Thorlabs LED 590 nm. *I*_exc_ = 69.2 mW cm^−2^ and (**c**) Excitation with Thorlabs LED 470 nm. *I*_exc_ = 94.2 mW cm^−2^.

The photocycle dynamics and the photo-degradation dynamics of CaRh samples were studied. In the photocycle experiments the samples were excited for a short time interval and attenuation coefficient spectra were measured before, during, and after light exposure. Temporal absorption changes during light exposure and after light exposure were recorded at fixed probe wavelengths with a time resolution of *t*_res_ = 0.0125 s (time step interval). For photo-degradation studies the samples were excited in several repetitions over long time periods with short recovery periods in between. In the photocycle and the photo-degradation experiments, the samples were excited at three different spectral positions around 590 nm (with Thorlabs LED 590 nm), 530 nm (with Thorlabs LED 530 nm), and 470 nm (with Thorlabs LED 470 nm). The spectral distributions of the light emitting diodes are indicated in Figure 7.

#### 2.3.1. Photocycle Dynamics of CaRh

**Figure 8:**
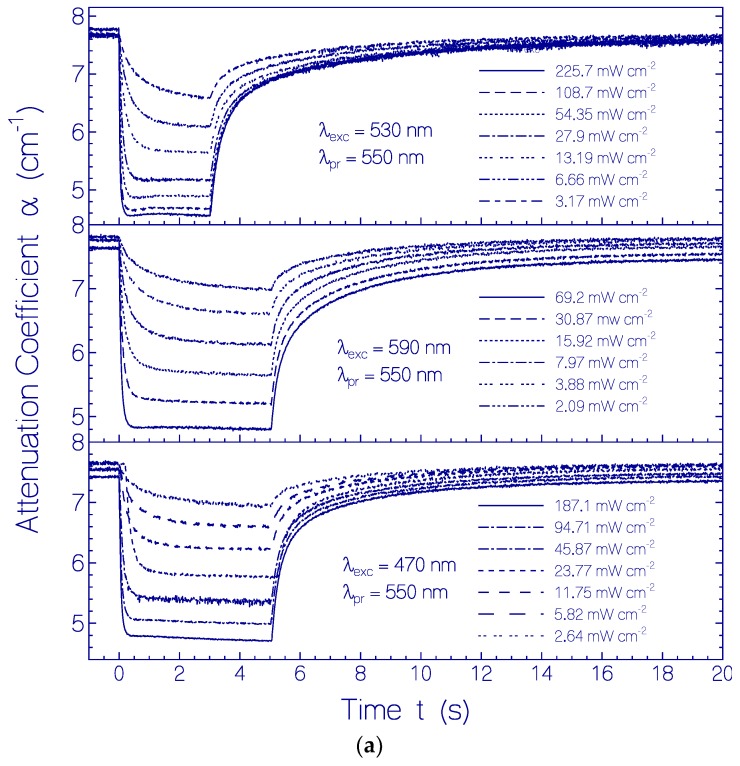

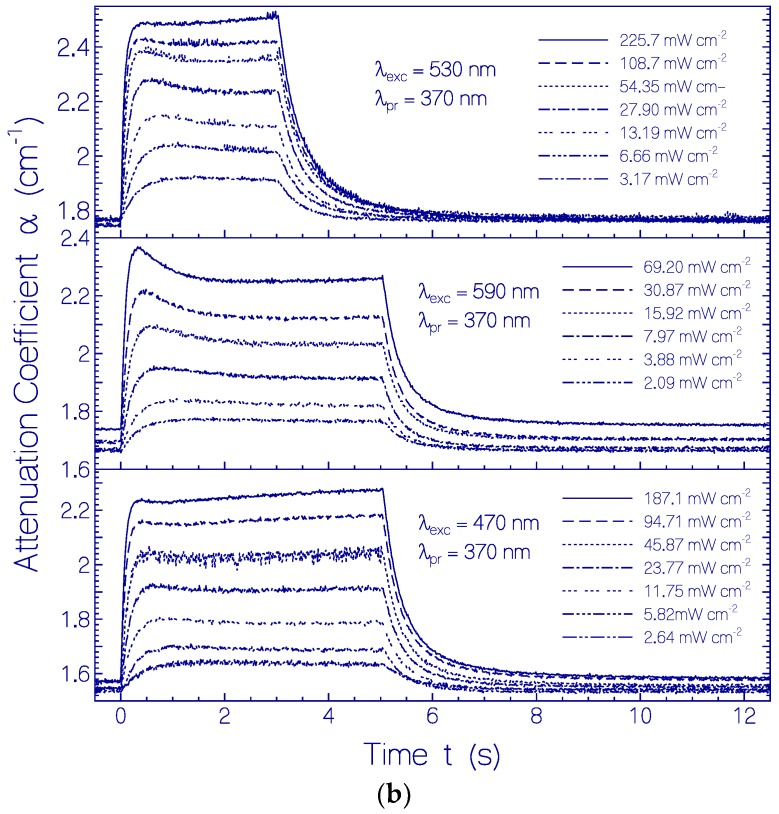
Temporal attenuation coefficient development of CaRh in pH 7.3 HEPES/MOPS buffer at (**a**) *λ*_pr_ = 550 nm and (**b**) *λ*_pr_ = 370 nm before, during, and after photo-excitation with LED 530 nm (top parts), LED 590 nm (middle parts), and LED 470 nm (bottom parts). The applied input excitation intensities *I*_exc_ are listed in the sub-figures.

**Figure 9:**
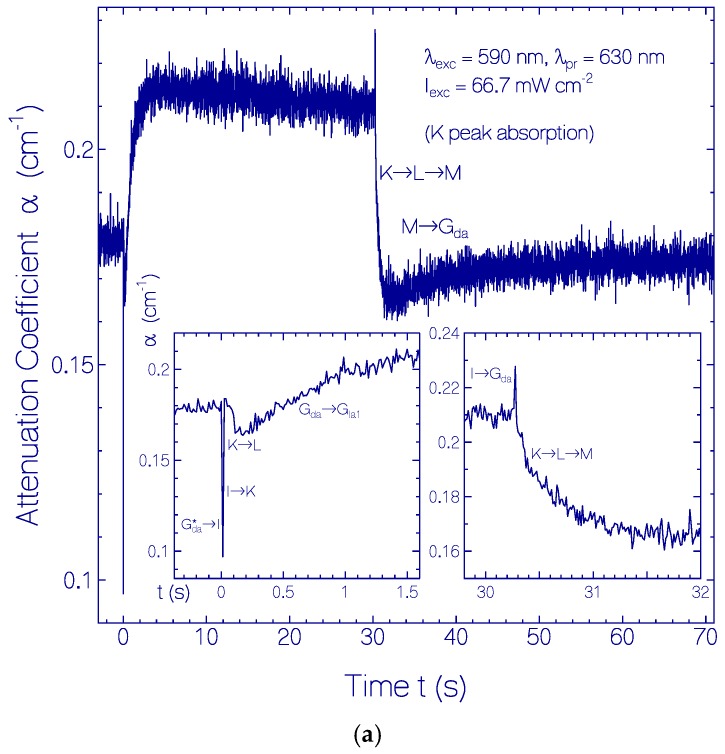

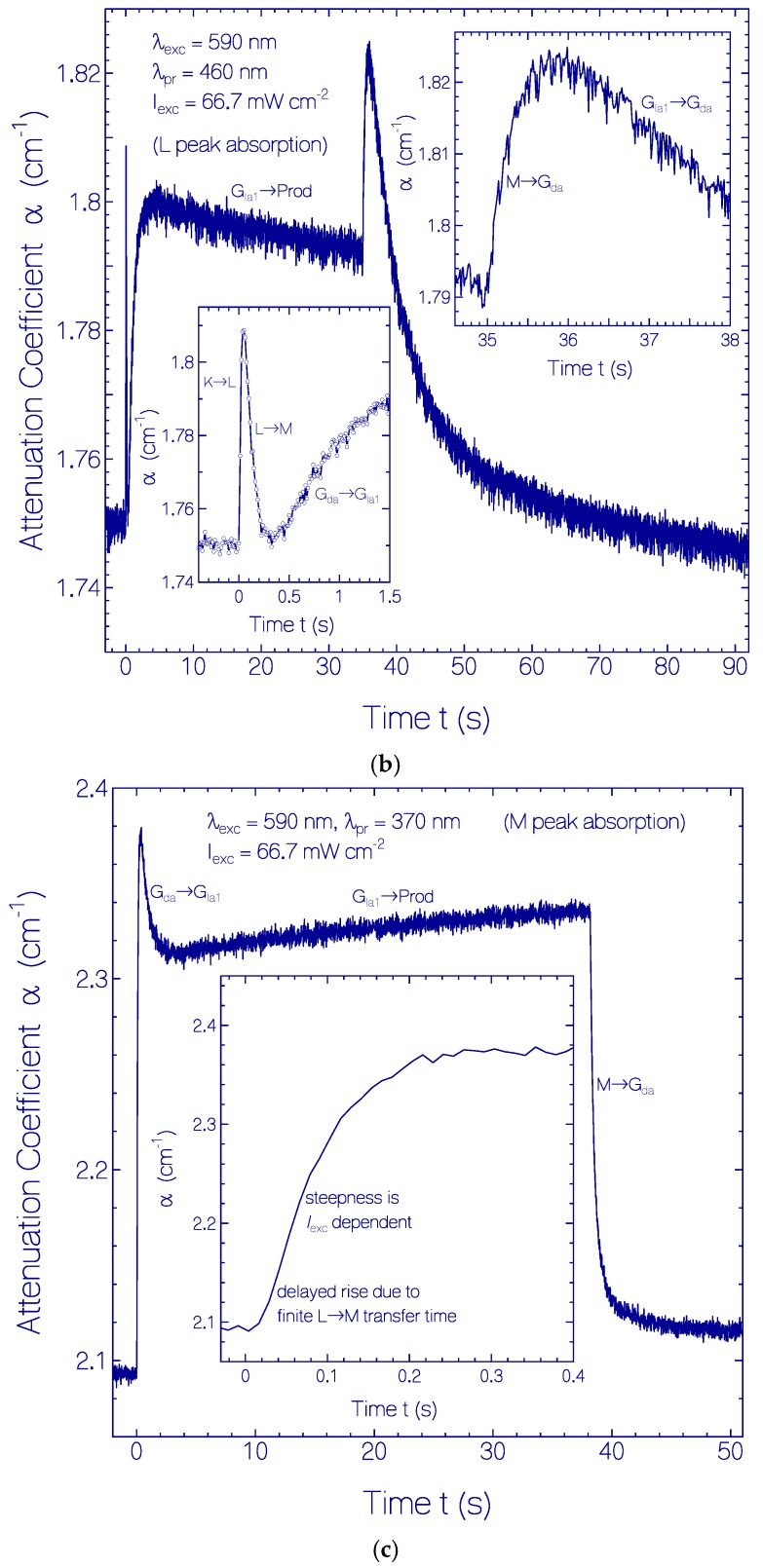

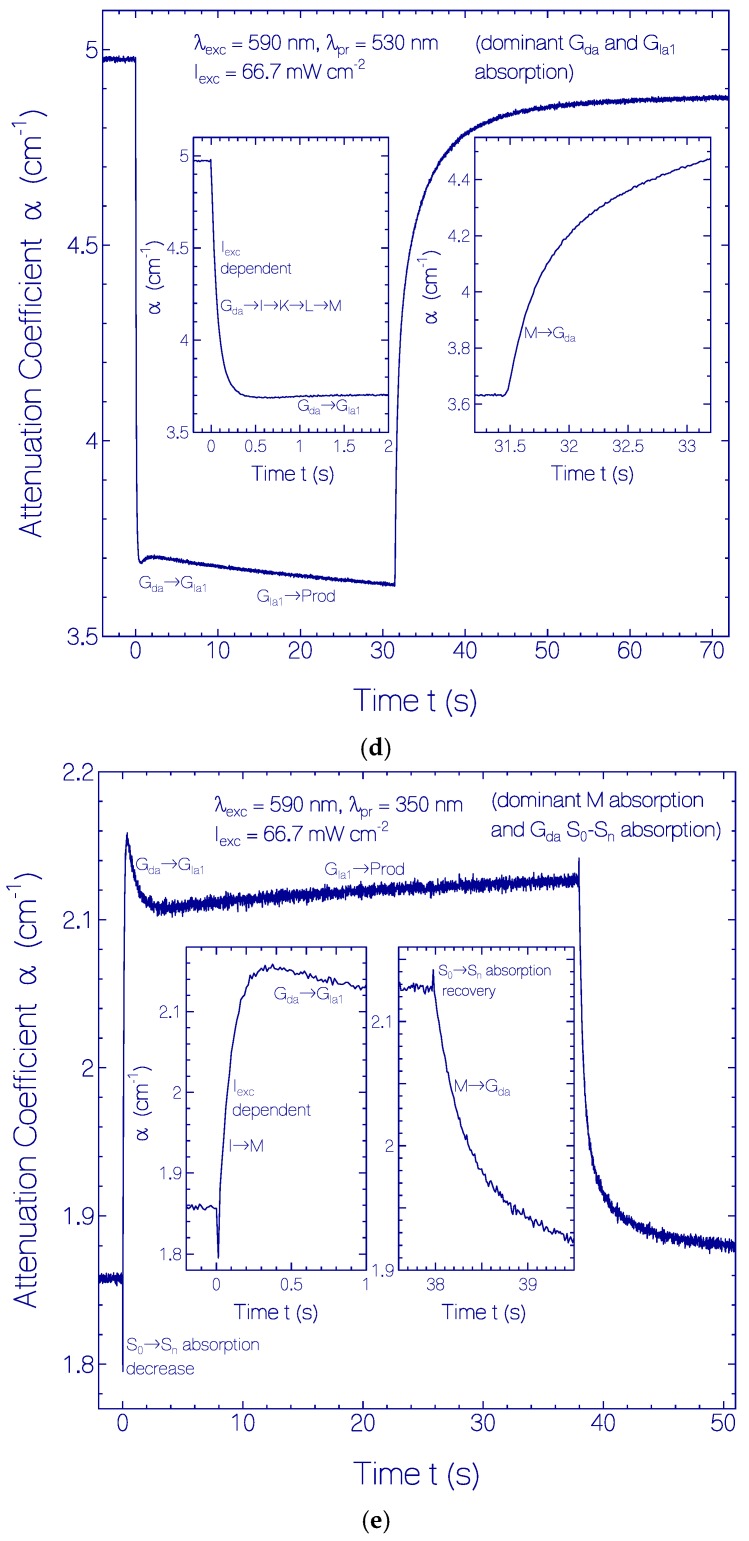
Temporal attenuation coefficient development of CaRh in pH 7.3 HEPES/MOPS buffer at (**a**) *λ*_pr_ = 630 nm (approximate peak absorption position of PRSB_13*-cis*_ K intermediate); (**b**) *λ*_pr_ = 460 nm (approximate peak absorption position of PRSB_13*-cis*,cirp_ L intermediate); (**c**) *λ*_pr_ = 370 nm (approximate peak absorption position of RSB_13*-cis*_ M intermediate); (**d**) *λ*_pr_ = 530 nm (dominant overlap of PRSB_all*-trans*_ G_da_ dark-adapted absorption band and G_la1_ light-adapted absorption band) and (**e**) *λ*_pr_ = 350 nm (dominant overlap of RSB_13*-cis*_ M absorption band and ground-state to higher excited-state G_da_ dark-adapted absorption band).

The photocycle results for excitation with LED 530 nm, LED 590 nm and LED 470 nm are presented in Figure 7, Figure 8 and Figure 9 and Figure S4.

For the absorption coefficient spectra presented in Figure 7a the CaRh sample was excited with Thorlabs LED 530 nm. The spectral light distribution of the LED 530 nm is included in Figure 7a. The input excitation intensity was *I*_exc_ = 226 mW cm^−2^ and the duration of light exposure was *t*_exc_ = 12 s. Within *t*_exc_ = 3 s a new absorption band (this band is named Rh-365) in the violet and near ultraviolet spectral region was formed and the original first absorption band (named Rh-541) in the green spectral region was lowered, slightly blue shifted and spectrally broadened (this shifted band is named Rh-527). After *t*_exc_ = 12 s the excitation was switched off, and the sample recovery was observed over a time range of 102 s. After excitation light switch-off the absorption coefficient spectrum recovered dominantly back to the situation before light exposure. A complete recovery did not occur because of some permanent photo-product formation.

The photocycle behavior of CaRh in the case of sample excitation with Thorlabs LED 590 nm and Thorlabs LED 470 nm are displayed in Figure 7b,c, respectively. The qualitative behavior was similar to the excitation with Thorlabs LED 530 nm (Figure 7a).

The temporal attenuation coefficient development of the investigated CaRh samples at *λ*_pr_ = 550 nm before, during, and after photo-excitation with LED 530 nm, LED 590 nm and LED 470 nm for various excitation intensities *I*_exc_ is shown in Figure 8a. The situation for *λ*_pr_ = 370 nm is shown in Figure 8b. Light excitation occurred in the time range of 0 ≤ *t* ≤ 3 s for LED 530 nm and in the time range of 0 ≤ *t* ≤ 5 s for LED 590 nm and LED 470 nm. The steepness of attenuation coefficient changes at the start of light excitation increased with excitation intensity. Attenuation coefficient plateaus are formed. The attenuation coefficient changes approach limits with increasing excitation intensity (complete conversion of the dark-adapted CaRh to the light-adapted CaRh, see also Supplementary Material S5 with Figure S4a,b). After excitation light switch-off the attenuation coefficients recovered nearly fully back to the situation before light exposure.

In order to gain information on the photocycle dynamics of CaRh (see Discussion below) the temporal attenuation coefficient development of CaRh was measured in the probe wavelength region from *λ*_pr_ = 300 to 630 nm in steps of 10 nm. The sample was exposed using LED 590 nm with *I*_exc_ = 66.7 mW cm^−2^ over a time range of *t_exc_* between 30 and 40 s. The attenuation coefficient development before, during and after exposure was followed over a time range of 150 s. The time resolution was *t*_res_ = 0.0125 s. The α(*λ*_pr_,*t*) curves indicated the build-up and decay of intermediate absorption bands centered around ≈630 nm (Rh-630), ≈460 nm (Rh-460), ≈365 nm (Rh-365), and ≈527 nm (Rh-527). As examples, the temporal attenuation coefficient developments at the probe wavelengths *λ*_pr_ = 630, 460, 370, 530 and 350 nm are shown in Figure 9a–e, respectively. The curves show transient temporal structures at light switch-on and light switch-off. These time dependences were used to develop schemes of the primary photocycle dynamics of initially dark-adapted CaRh (Rh-541) and of the secondary photocycle dynamics of CaRh under light-adapted conditions (Rh-527). They will be discussed below in the Discussion part.

#### 2.3.2. Photo-Degradation Dynamics of CaRh

Continued CaRh sample excitation with LED 590 nm, LED 530 nm, or LED 470 nm after light-adapted state formation caused photo-induced CaRh photo-degradation (CaRh_Prod_ photoproduct formation).

**Figure 10:**
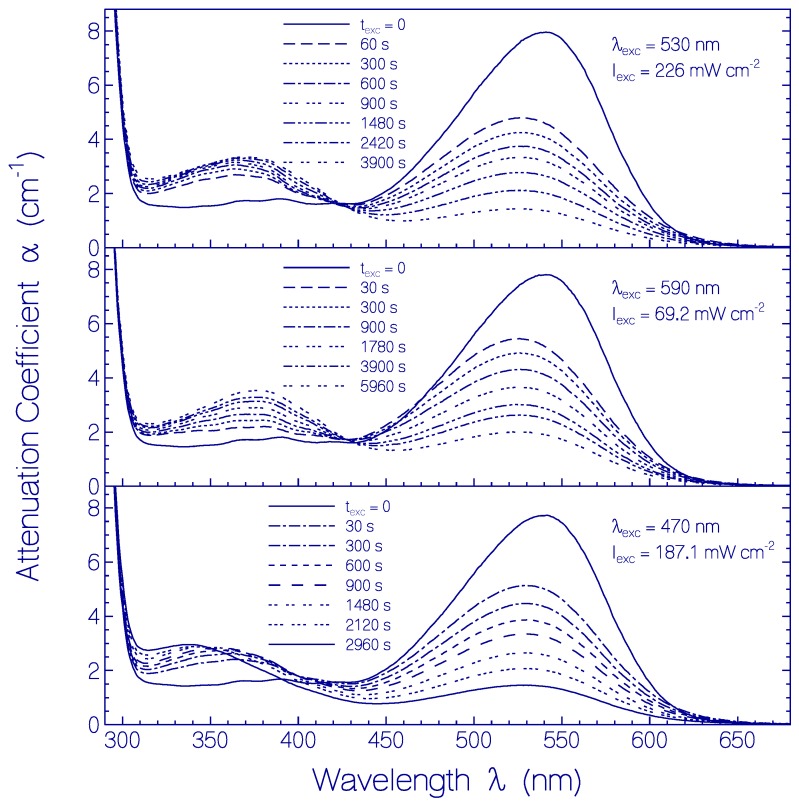
Development of attenuation coefficient spectra of CaRh in pH 7.3 HEPES/MOPS buffer in the light-adapted state during light exposure with LED 530 nm (top part), LED 590 nm (middle part), and LED 470 nm (bottom part). Input excitation intensities *I*_exc_ and durations of light exposure *t*_exc_ are listed in the sub-figures.

This situation is shown in Figure 10 for long-time exposure of samples with LED 530 nm (top part, *I*_exc_ = 226 mW cm^−2^), LED 590 nm (middle part, *I*_exc_ = 69.2 mW cm^−2^), and LED 470 nm (bottom part, *I*_exc_ = 187.1 mW cm^−2^). The inhomogeneous S_0_–S_1_ absorption band of CaRh in the light-adapted state (Rh-527) decreased with exposure time and the absorption in the violet and near ultraviolet spectral range increased and changed its shape due to photoproduct (CaRh_Prod_) formation.

**Figure 11:**
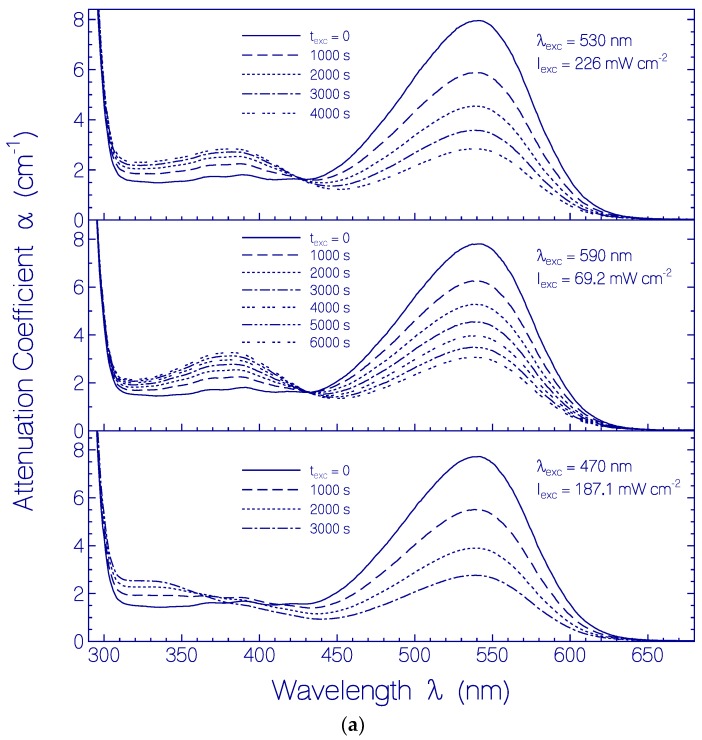

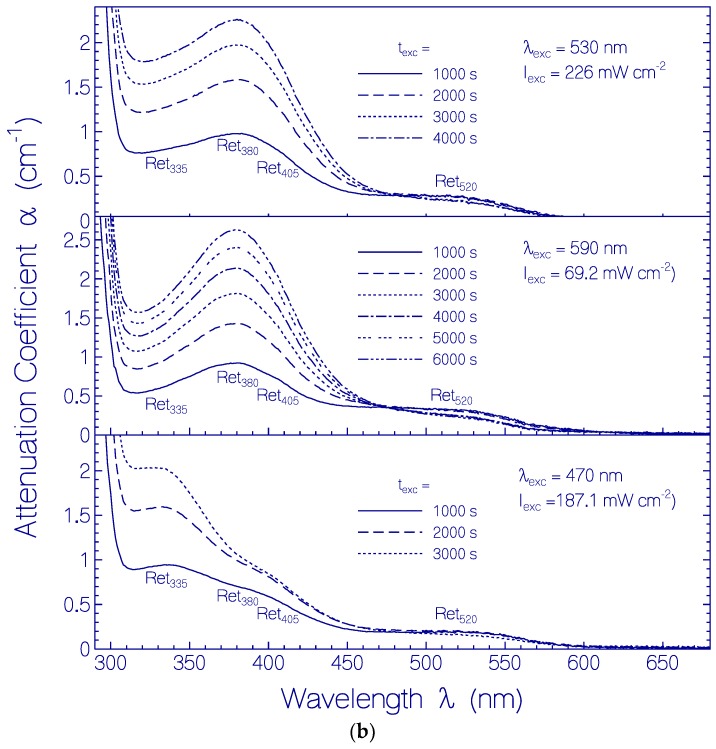
(**a**) Attenuation coefficient spectra of dark-adapted CaRh_da_ in pH 7.3 HEPES/MOPS buffer before light exposure (*t*_exc_ = 0) and after light exposures with LED 530 nm (top part), LED 590 nm (middle part), and LED 470 nm (bottom part); (**b**) Attenuation coefficient spectra of formed photoproducts of CaRh in pH 7.3 HEPES/MOPS buffer due to light exposure with LED 530 nm (top part), LED 590 nm (middle part), and LED 470 nm (bottom part). Input excitation intensities *I*_exc_ and durations of light exposure *t*_exc_ are listed in the sub-figures of (**a**) and (**b**).

The permanent spectral changes due to long-time exposure of CaRh samples are seen in Figure 11a where attenuation coefficient spectra of dark-adapted CaRh samples are shown before light exposure (*t*_exc_ = 0) and in cases of recovery in the dark after continued exposure in repeated intervals of 1000 s. For *λ* > 430 nm the decrease of absorption of the S_0_–S_1_ transition of Rh-541 due to photo-degradation is seen, and for *λ* < 430 nm absorption changes due to photo-product formation (CaRh_Prod_) are seen.

The attenuation coefficient spectra development of the photoproducts is seen in Figure 11b where the attenuation coefficient contribution of dark-adapted CaRh in Figure 11a is subtracted. In the three cases of excitation with LED 530 nm, LED 590 nm and LED 470 nm the formation of (at least) four photoproducts, Ret_520_, Ret_405_, Ret_380_ and Ret_335_, is revealed. Their absorption peaks are at ≈ 520 nm (Ret_520_), ≈ 405 nm (Ret_405_), ≈ 380 nm (Ret_380_), and ≈ 335 nm (Ret_335_). Ret_520_ is thought to be a protonated retinal Schiff base form. It may have lost its proper covalent binding to the opsin protein. Ret_520_ absorbs in the spectral region of the LED 590 nm, LED 530 nm, and LED 470 nm excitation light sources. Therefore it is weakly photo-degraded (its amount slightly decreases) with long-time exposure. Ret_405_ and Ret_380_ are thought to be deprotonated retinal Schiff base conformations. Their proper covalent binding to the opsin protein may have been lost. Ret_335_ may be a deprotonated retinol bound to the opsin protein or released from it. The absorption spectrum of Ret_335_ agrees with the absorption spectral shape of retinol [25]. The long-wavelength absorption tails of Ret_405_ and Ret_380_ overlap with the excitation spectrum of LED 470 nm and therefore Ret_405_ and Ret_380_ seem to be partly photo-degraded to Ret_335_ in the case of long-time LED 470 nm exposure.

The quantum yield of photo-degradation Φ_d_ of CaRh in its light-adapted state (Rh-527) is determined by the absorption decrease of CaRh in its dark-adapted state (Rh-541) of Figure 11a at *λ* = 541 nm due to excitation photon absorptions around 596 nm (LED 590 nm), 520 nm (LED 530 nm) and 462 nm (LED 470 nm) of light-adapted CaRh in Figure 10. The calculation of Φ_d_ from the experimental curves in Figure 10 and Figure 11a is given in the Supplementary Material S6.

**Figure 12:**
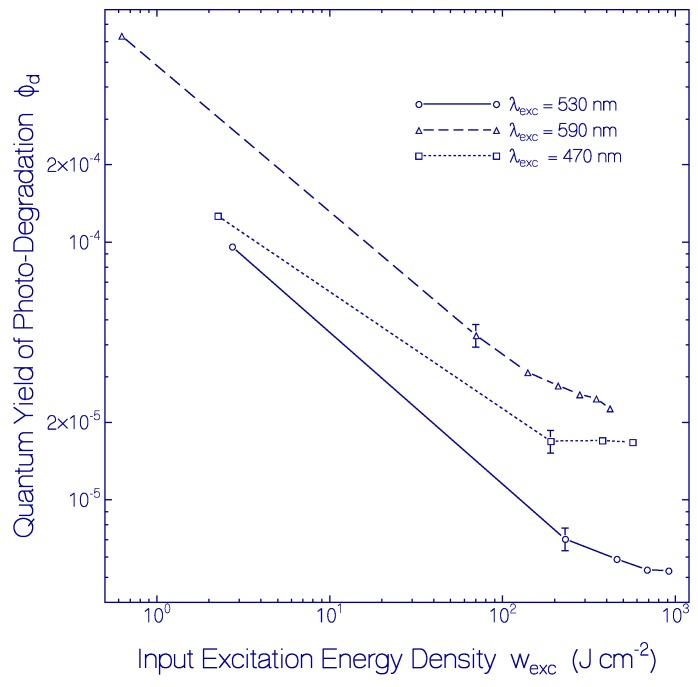
Quantum yields of photo-degradation of CaRh in pH 7.3 HEPES/MOPS buffer versus accumulated input excitation energy density w_exc_ for sample exposure with LED 590 nm (triangles), LED 530 nm (circles), and LED 470 nm (squares). Data of Figure 10 and Figure 11a were applied in the Φ_d_ calculation.

The obtained quantum yields of photo-degradation Φ_d_ of CaRh versus accumulated input excitation energy density w_exc_ = $\int I_{exc}dt$ for sample excitation with LED 530 nm, LED 590 nm and LED 470 nm are displayed in Figure 12. Φ_d_ decreased with exposure time (accumulated input excitation energy density). Some saturation is obsvered for very long-time sample exposure (w_exc_ > 100 J cm^−2^). The efficiency of photo-degradation of light-adapted CaRh also depended on the excitation wavelength. It was highest for excitation with LED 590 nm (Φ_d_(w_exc_ = 0.62 J cm^−2^) = 6.3 × 10^−4^, Φ_d_(w_exc_ = 416 J cm^−2^) = 2.3 × 10^−5^), in between for excitation with LED 470 nm (Φ_d_(w_exc_ = 2.3 J cm^−2^) = 1.3 × 10^−4^, Φ_d_(w_exc_ = 564 J cm^−2^) = 1.7 × 10^−5^), and lowest for for excitation with LED 530 nm (Φ_d_(w_exc_ = 2.7 J cm^−2^) = 9.6 × 10^−5^, Φ_d_(w_exc_ = 910 J cm^−2^) = 5.3 × 10^−6^). This excitation wavelength and excitation energy density dependence of CaRh photo-degradtion indicates an inhomogeneous nature of the CaRh protein concerning the excitation wavelength dependence (higher stability around wavelength position of maximum absorption) and the exposed excitation energy density (less stable protein fraction photo-degrades first).

## 3. Discussion

The rhodopsin-guanylyl cyclase CaRhGC from the nematophagus fungus *Catenaria anguillulae* belongs to the class of enzymerhodopsins of microbial proteins which consist of a rhodopsin domain and an enzyme domain [8,9,26,27,28]. Light excitation of the rhodopsin domain results in the activation of the guanylyl cyclase domain and causes the conversion of GTP (guanosine triphosphate) to cGMP (cyclic guanosine monophosphate).

The rhodopsin-guanylyl cyclase CaRhGC from *Catenaria anguillulae* was expressed recently and its light-activated guanylyl cyclase activity was demonstrated [4]. Previously, another rhodopsin-guanylyl cyclase BeRhGC from the aquatic fungus *Blastocladiella emersonii* was studied and applied as optogenetic tool in [4,29,30]. An absorption and emission spectroscopic characterization of the rhodopsin domain BeRh of BeRhGC was carried out in [6] (named Rh (BE) of RhGC in [6]). Here, an absorption and emission spectroscopic characterization of the rhodopsin part CaRh of CaRhGC was carried out and and its behavior is compared below with BeRh and with channelrhodopsin ChR2 from *Chlamydomonas reinhardtii*.

### 3.1. Behavior of CaRh from Catenaria anguillulae

#### 3.1.1. Spectral and Thermal Studies

The rhodopsin CaRh is thermally very stable. The apparent protein melting temperature of CaRh is *ϑ*_m_ = 62 ± 2 °C. For CaRh at 3.5 °C no attenuation coefficient rise in the transparency region (*λ* > 640 nm) was observered within the investigation period of 103 days. It occurred an attenuation coefficient spectrum reduction due to CaRh aggregate cluster compactization with storage time [24]. At room temperature (20.5 °C) some continuous attenuation coefficient rise in the transparency region was observed within the first 144 h due to protein aggregation, then the attenuation coefficient decreased because of protein aggregate sedimentation. The higher the apparent protein melting temperature *ϑ*_m_ the longer is the protein melting time *t*_m_ at the temperature of experimental investigation of the protein (e.g., room temperature). The protein melting time or half-time *t*_m_ is defined as the time duration of unfolding of 50% of the protein [21]. The protein melting time of CaRh at *ϑ* = 3.5 °C was longer than the time of experimental observation of 103 days (during this time no measurable increase of light scattering, see Figure 5). Also at *ϑ* = 20.5 °C the melting time of CaRh was longer than the time of experimental observation of 312 h (scattering coefficient α_s_(750 nm, 312 h) ≈ 0.25 cm^−1^ in Figure 6 compared to α_s_(750 nm, 69.2 °C) ≈ 5 cm^−1^ in Figure 4a).

Fresh thawed CaRh exhibits a smooth inhomogeneous broadened S_0_–S_1_ absorption band and shows the structure of less inhomogeneous broadened higher excitation bands (S_0_–S_2_ and S_0_–S_3_ transitions, see Figure 1). If the absorption coefficient spectrum of fresh CaRh in the wavelength range from 440 to 310 nm would belong to S_0_–S_1_ transitions of different retinal isomers, then another photocycle behavior in this spectral range would be expected than observed in Figure 7. Inhomogeneous absorption line broadening means the presence of a distribution of species with shifted absorption spectra [31,32]. Here it indicates the presence of a distribution of retinal and opsin protein conformations with differing retinal—ospin interactions causing a distribution of ground-state and excited-state singlet potential energy surfaces.

The inhomogeneous nature of the S_0_–S_1_ absorption band of CaRh shows up in the variation of fluorescence quantum distributions (Figure 2a,b) and the fluorescence quantum yield (Figure 3) with fluorescence excitation wavelength within the S_0_–S_1_ absorption band (*λ*_F,exc_ ≥ 440 nm). The isomerization path in the retinal S_1_ potential energy surface depends on the excitation wavelength (locally excited state LE, see Figure S5 in Supplementary Material S8). S_1_–S_0_ fluorescence emission along the S_1_ potential energy surface relaxation path towards the S_1_ state funnel Fu (conical intersection [14], position of S_1_–S_0_ twisted internal conversion [15]) determines the excitation wavelength dependent fluorescence emission quantum distribution and fluorescence quantum yield. The small fluorescence quantum yield indicates a barrierless S_1_-state potential energy surface relaxation.

Higher excited state S_0_–S_2_ and S_0_–S_3_ transitions of PRSB in CaRh turned out to be less inhomogeneous broadened showing some vibronic structure. They follow higher excited state isomerization paths with activation barriers to funnel positions indicated by structured fluorescence emissions and higher fluorescence quantum yields.

#### 3.1.2. Photocycle Studies

Generally photo-excitation of rhodopsins causes retinal spatial *cis*-*trans* isomerization [8]. The photo-isomerization of protonated retinal Schiff base PRSB in rhodopsins often leads to a deprotonated retinal Schiff base intermediate RSB in the photocycle process [33,34,35,36]. The absorption coefficient spectra development of CaRh during and after photo-excitation displayed in Figure 7 indicates the partial formation of deprotonated retinal Schiff base intermediate RSB (Rh-365) and protein reconformation changing the orignal protonated retinal Schiff base PRSB (Rh-541) to intermediate protonated retinal Schiff base PRSB (Rh-527). The photo-excitation intensity dependent temporal attenuation coefficient development of CaRh depicted in Figure 8a,b and Figure S4a,b indicates that opsin reconformation hinders protonated retinal Schiff base deprotonation in the Rh-527 photo-isomerization cycle causing only low-efficient Rh-527 photo-degradation (CaRh_Prod_ formation).

The time-resolved attenuation coefficient developments at selected probe wavelengths in the photocycle studies shown in Figure 9a–e reveal the formation of isomer intermediates (S_0_ transition state intermediate I, Rh-630, and Rh-460) in the photo-isomerization dynamics of CaRh.

The experimental photocycle studies lead to the following interpretation: (i) the photo-excitation of dark-adapted CaRh_da_ causes a primary all*-trans*—13*-cis* photo-isomerization cycle with protonated retinal Schiff base to deprotonated retinal Schiff base conversion (light-adapted CaRh_la2_ formation, Rh-365); (ii) it involves an all*-trans* back photo-isomerization and protein restructuring cycle changing CaRh_da_ to a light-adapted ground-state conformation CaRh_la1_ (Rh-527); (iii) photo-excitation of CaRh_la1_ causes a secondary all*-trans*—13*-cis* photo-isomerization cycle without protonated retinal Schiff base deprotonation. (Structural formulae of PRSB_all*-trans*_, PRSB_13*-cis*_, and RSB_13*-cis*_ are shown in Figure S2 of [6]).

**Figure 13:**
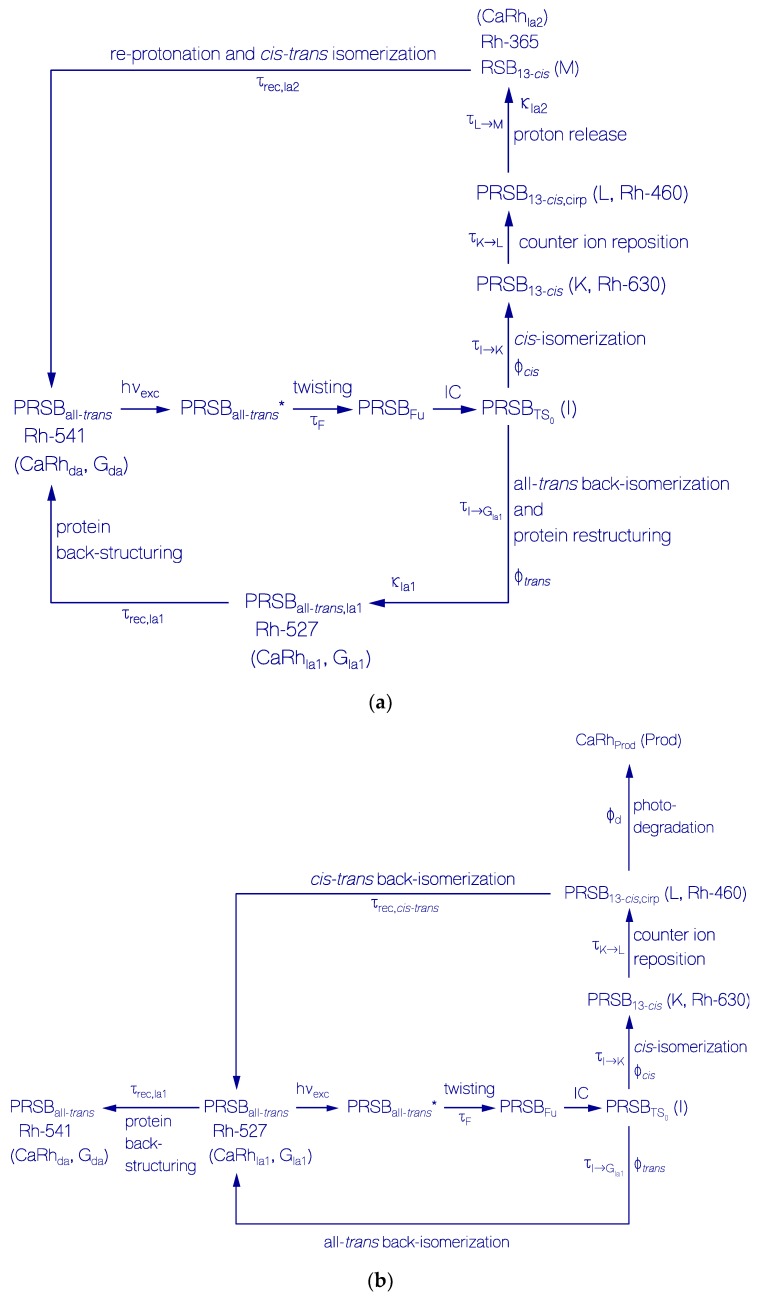
(**a**) Scheme of primary photocycle dynamics (upper part) and all-*trans* back-isomerization with opsin restructuring cycle (lower part) of CaRh in pH 7.3 HEPES/MOPS buffer; (**b**) Scheme of secondary photocycle dynamics including photo-degradation, all-*trans* back-isomerization and light-adapted (la1) to dark-adapted (da) ground-state relaxation of CaRh in pH 7.3 HEPES/MOPS buffer.

The proposed photocycle schemes are displayed in Figure 13a (primary photocycle, and all*-trans* back-isomerization with protein restructuring cycle) and Figure 13b (secondary photocycle including photo-degradation). Schematic reaction coordinate diagrams for the primary photocycle including protein restructuring of initially dark-adapted CaRh and the secondary photocycle of light-adapted CaRh without deprotonation are shown in Figures S5 and S6 of Supplementary Material S8, respectively. In the following the primary all*-trans*—13*-cis* photocycle scheme of Figure 13a and Figure S5 is explained first, then the all*-trans* back-isomerization and protein restructuring cycle of Figure 13a and Figure S5 is described, and then follows a description of the secondary photocycle scheme of Figure 13b and Figure S6. After that relevant photocycle parameters are extracted from the experimental results (Table 2 and Table 3).

##### Primary All*-trans*—13*-cis* Photocycle of Initially Dark-Adapted CaRh

The proposed all*-trans*—13*-cis* photocycle scheme of initially dark-adapted CaRh (named CaRh_da_) is shown in Figure 13a (upper part) and Figure S5. The retinal in the CaRh_da_ dark-adapted state (also named G_da_ for dark-adapted ground-state and Rh-541 considering its first peak absorption wavelength position) is thought to be all*-trans* protonated retinal Schiff base PRSB_all*-trans*_ [6,8]. Photo-excitation of PRSB_all*-trans*_ to a locally excited electronic state PRSB_all-trans_* (LE) starts photo-isomerization by relaxation (twisting) along the excited state potential energy surface to a funnel position PRSB_Fu_ (Fu, conical intersection position, twisted internal conversion position). The relaxation time constant from locally excited state LE to funnel Fu is experimentally given by the fluorescence lifetime *τ*_F_ in the sub-picosecond region for barrier-less relaxation to the picosecond region for barrier-slowed down relaxation (see Table 1). It occurs internal conversion (IC) from the funnel position Fu to a transition state position TS_0_ (${PRSB}_{{TS}_{0}}^{}$) on the S_0_ potential energy surface. This state is labled I. Relaxation out of the ${PRSB}_{{TS}_{0}}^{}$ labile transition state position I leads to a branching of relaxation along a *cis* isomerization paths (quantum yield of *cis*-isomerization Φ*_cis_*) and along the all*-trans* back-isomerization path (quantum yield of all*-trans* back-isomerization Φ*_trans_* = 1 − Φ*_cis_*). The *cis* isomerization path leads to the formation of PRSB_13*-cis*_ (Rh-630, named K intermediate [5] following the bacteriorodospin photocycle nomencature [36]). PRSB_13*-cis*_ relaxes to PRSB_13*-cis*,cirp_ (Rh-460, L intermediate [5,36]) by counter ion repositioning. PRSB_13*-cis*,cirp_ relaxes to RSB_13*-cis*_ (Rh-365, M intermediate [5,36], light-adapted CaRh_la2_) by proton release. RSB_13*-cis*_ recovers back to PRSB_all*-trans*_ (CaRh_da_, G_da_, Rh-541) by re-protonation and *cis-trans* isomerization with time-constant *τ*_rec,la2_.

##### Photo-Induced all*-trans* Back-Isomerization and Opsin Restructuring Cycle

The photo-excitation of CaRh_da_ (PRSB_all*-trans*_) causes besides the all*-trans*—13*-cis* photo-isomerization cycle an all*-trans* back-isomerization and opsin protein restructuring cycle generating light-adapted ground-state CaRh_la1_ (also named G_la1_, Rh-527, PRSB_all*-trans*,la1_). CaRh_la1_ recovers back to CaRh_da_ by protein back-structuring with time-constant *τ*_rec,la1_. The all*-trans* back-isomerization and protein restructuring photocycle is included in Figure 13a (lower part) and Figure S5.

##### Secondary Photocycle of Light-Adapted CaRh_la1_

The secondary photo-isomerization cycle of CaRh_la1_ is illustrated in Figure 13b and Figure S6. As in the primary photocycle, photo-excitation of PRSB_all*-trans*,la1_ leads to metastable transition state ${PRSB}_{{TS}_{0},la1}^{}$ (I intermediate) formation from where all*-trans* back-isomerization and all*-trans*—13*-cis* isomerization occurs. The *cis* isomerization causes PRSB_13*-cis*,la1_ (K, Rh-630) formation. Counter ion repositioning changes PRSB_13*-cis*,la1_ to PRSB_13*-cis*,cirp,la1_ (L, Rh-460). Contrary to the primary photo-isomerization cycle of CaRh_da_ no reversible deprotonation of PRSB_13*-cis*,cirp,la1_ takes place due to the protein restructuring. Instead PRSB_13*-cis*,cirp,la1_ recovers back to PRSB_all*-trans*,la1_ by 13*-cis*—all*-trans* back-isomerization with time constant *τ*_rec,*cis-trans*_. The back-isomerization with time constant *τ*_rec,*cis-trans*_ has to be short compared to the protein back-structuring time constant *τ*_rec,la1_ (i.e., *τ*_rec,*cis-trans*_ << *τ*_rec,la1_) since only weak population accumulation of Rh-460 is observed (only rise of attenuation coefficient at 460 nm from 1.75 to 1.8 cm^−1^ due to 590 nm light exposure in Figure 9b). The photo-excitation of CaRh_la1_ causes some irreversible degradation to CaRh_Prod_ photoproducts (Prod, quantum yield of photo-degradation Φ_d_, for photoproduct characterization see above).

##### Photocycle Parameters

The photo-excitation of CaRh_da_ causes only a partial conversion of CaRh_da_ to CaRh_la2_ because of the parallel back-isomerization and protein restructuring photocycle of CaRh_da_ to CaRh_la1_ conversion.

The limiting fraction ĸ_la1_ of excited CaRh_da_* converted to CaRh_la1_ at high excitation intensity is obtained from the ratio of the absorption strength of the S_0_–S_1_ transition of CaRh_la1_ at high excitation intensity (dashed curves in Figure 7 for *t*_exc_ = 3 s) to the initial absorption strength of the S_0_–S_1_ transition of CaRh_da_ before excitation (solid curves in Figure 7). The analysis presented in the Supplementary Material S7 gives ĸ_la1_ ≈ 0.73 (ĸ_la1_ is included in Table 3). The limiting fraction ĸ_la2_ of excited CaRh_da_* converted to Ca Rh_la2_ is ĸ_la2_ = 1 − ĸ_la1_ ≈ 0.27 (ĸ_la2_ is included in Table 2).

The initial quantum yield of all-trans—13-cis photo-isomerization Φ_cis_ (Figure S5) of CaRh_da_ is deduced from the initial light induced absorption change at λ_pr_ = 550 nm of middle part of Figure 8a for λ_exc_ = 590 nm and t_exc_ = 0.0125 s. The result determined in the Supplementary Material S7 is Φ_cis_ = 0.46 ± 0.05. The quantum yield of all-trans back-isomerization is Φ_trans_ = 1 − Φ_cis_ = 0.54 ± 0.05.

Time constants of the photocycle intermediate formations and decays are extracted from the temporal attenuation coefficient developments of Figure 9a–e. The obtained parameters are collected in Table 2 for the CaRh primary retinal photocycle dynamics, and in Table 3 for the CaRh secondary retinal photo-isomerization and protein restructuring photocycle dynamics.

In Figure 9a the transient attenuation coefficient development at *λ*_pr_ = 630 nm is shown where the K intermediate (PRSB_13*-cis*_) has its absorption peak. The sharp attenuation dip in Figure 9a at *t* = 0 is due to G_da_* → I relaxation. The time constant of G_da_* → I relaxation is expected to be on the sub-picosecond to picosecond time scale ($\tau_{G_{da}^{*}\to I}\geq \tau_{F}$). This dip disappears within the time resolution step of 0.0125 s due to I → K *cis* isomerization (*τ*_I→K_ < *t*_res_ = 0.0125 s) and I → G_la1_ *trans* back-isomerization ($\tau_{I\to G_{la1}}$ < *t*_res_ = 0.0125 s). The following slight attenuation decrease seen in the left inset of Figure 9a is due to conversion of K to L. The time constant is *τ*_K→L_ = 0.048 ± 0.005 s. The further rise of α(630 nm) is due to conversion of G_da_ to G_la1_. Its build-up time is equal to the G_la1_ to G_da_ recovery time. The obtained time constant is $\tau_{G_{la1}\to G_{da}}$ = *τ*_rec,la1_ = 0.8 ± 0.1 s. At light switch-off CaRh recovers to the dark-adapted situation. The spike at the moment of light switch-off is due to I → G_da_ conversion. The following absorption decrease is caused by K → L → M conversion. The final slow absorption rise is thought to be mainly due to M to G_da_ recovery.

In Figure 9b the transient attenuation coefficient development at *λ*_pr_ = 460 nm is shown where the L intermediate (PRSB_13*-cis*,cirp_) has its absorption peak. The attenuation peak at the onset of light exposure (shutter opening time constant 1 ms) is due to K → L counter ion repositioning (PRSB_13*-cis*_ → PRSB_13*-cis*,cirp_, absorption build-up at 460 nm) and L → M proton release (PRSB_13*-cis*,cirp_ → RSB_13*-cis*_, absorption decrease at 460 nm). The time constant of L → M conversion is determined from the left inset of Figure 9b to be *τ*_L→M_ = 0.123 ± 0.005 s. The following rise of absorption is caused by the conversion of G_da_ to G_la1_. It follows a slight decrease of α(460 nm) because of G_la1_→Prod photoproduct formation. At light switch-off the absorption rise is caused by M → G_da_ relaxation (time constant *τ*_rec,la2_). The following absorption decrease is thought to be due to G_la1_ → G_da_ recovery.

In Figure 9c, the transient attenuation coefficient development at *λ*_pr_ = 370 nm is displayed where the M intermediate (RSB_13*-cis*_, CaRh_la2_) has its absorption peak. The attenuation coefficient increase at the onset of light exposure has a slightly sigmoidal shape (delayed rise, see inset in Figure 9c) because of the delayed population of M in the I → K → L → M intermediate chain (*τ*_I→K_ < 0.0125 s, *τ*_K→L_ ≈ 0.048 s, *τ*_L→M_ ≈ 0.12 s). The steepness of the attenuation coefficient rise depends on the excitation intensity I_exc_ (increases with rising excitation intensity). The initially reached α(370 nm) attenuation peak decreases somewhat because of build-up of CaRh_la1_ (G_da_ → G_la1_ conversion) whose photo-excitation cycle does not involve M intermediate formation (time constant of attenuation decrease is given by *τ*_rec,la1_ by equilibration between G_da_ and G_la1_). The following slight rise of α(370 nm) is due to CaRh_la1_ → CaRh_Prod_ photo-degradation. After light swich-off attenuation coefficient α decreases because of M → G_da_ re-protonation and *cis-trans* isomerization (CaRh_la2_ recovery to CaRh_da_) with dominant time constant *τ*_rec,la2_ = 0.35 ± 0.01 s, and slower relaxation of the other intermediates with attenuation contribution at *λ*_pr_ = 370 nm to G_da_.

In Figure 9d, the transient attenuation coefficeint development at *λ*_pr_ = 530 nm is displayed where the absorption is dominated by the initial CaRh_da_ (G_da_) and the formed CaRh_la1_ (G_la1_). The initial absorption decrease after light switch-on is caused by G_da_ → I → K → L → M intermediate formation. The steepness of the decrease is I_exc_ dependent (sharper decrease for larger I_exc_). It follows a slight absorption increase due to G_la1_ formation with its secondary photo-isomerization cycle. The following slight attenuation coefficient decrease is due to photo-degradation G_la1_ → Prod. After light switch-off the attenuation coefficient recovers mainly because of M → G_da_ recovery.

In Figure 9e the transient attenuation development at *λ*_pr_ = 350 nm is displayed where the absorption is dominated by M (RSB_13*-cis*_) absorption as in the case of Figure 9c. The transient attenuation behavior is the same as in Figure 9c, only at the moment of light switch-on an additional attenuation dip and at the moment of light switch-off an additional attenuation spike are present. The dip and the spike are thought to be present because of G_da_ level depopulation (G_da_→I, dip) and G_da_ level repopulation (I→G_da_, spike) with associated S_o_–S_n_ absorption change.

### 3.2. Comparision of Behavior of CaRh from Catenaria anguillulae with Behavior of BeRh from Blastocladiella emersonii

The studied rhodopsin BeRh in [6] was thermally of low stability. The apparent protein melting temperature of BeRh was *ϑ*_m_ = 48.8 ± 2 °C. BeRh protein melting times of *t*_m_(1.65 °C) = 8.1 ± 0.2 day and *t*_m_(21.9 °C) = 1.45 ± 0.15 h were determined from the onset of strong light-scattering due to aggregation of unfolding proteins. For optogenetic studies the stability of the photoreceptor is crucial. Due to the increased protein stability of CaRh compared to BeRh, the application of CaRh is beneficial, in particular for experiments, which require a prolonged functionality of the photoreceptor, e.g., when repetitive illumination protocols over extended time periods are used.

Fresh thawed BeRh was composed of a mixture of retinal—protein conformations showing up in the rhodopsin absorption spectrum. In the inhomogeneous broadened absorption spectrum of BeRh the presence of (at least) four retinal isomers Ret_1, Ret_2, Ret_3, Ret_4 could be resolved (see Figure S3 of [6]). The retinal—protein conformation mixture also showed up in the fluorescence emission quantum distribution dependence on the fluorescence excitation wavelength (see Figure 2 in [6]). The retinal composition changed with storage time towards irreversible deprotonated (likely 13*-cis*) retinal Schiff base (Ret_4’) (see Figures 4a,b, S5 and S6 of [6]).

The photo-excitation dynamics of BeRh in the case of protonated retinal Schiff base PRSB_all*-trans*_ excitation (*λ*_exc_ = 532 nm) resulted in all*-trans*—13*-cis* photo-isomerization with subsequent retinal intermediate formations (see experimental curves in Figures 5–8 and schemes of Figures 9 and 10b of [6]). The photodynamics studies in [6] were carried out only at one excitation wavelength (second harmonic of cw Nd:YAG laser, *λ*_exc_ = 532 nm) with rather low excitation intensity (*I*_exc_ ≈ 16 mW cm^−2^ for Figure 5 and Iexc ≈ 22 mW cm−2 for Figures 6–8). The experimental photocycle/photo-degradation behavior of BeRh was found to be quite similar to that CaRh: for short-time exposure (*t*_exc_ = 0.1 s) a reversible photocycle behavior was observed (Figure 7a,b); the wavelength position of the first absorption maximum of Ret_1 shifted from *λ*_a,max_ = 527 nm in the dark-adapted state to *λ*_a,max_ = 518 nm in the light-adapted state (solid curve in Figure 5 for t_exc_ = 0 and dotted curve in Figure 5 for *t*_exc_ = 2.171 s); in the continued exposure over 990 s an attenuance plateau was reached within the first few seconds of excitation and then gradual irreversible photoproduct formation occurred (Figure 6).

The light-adapted Ret_1_la1_ (PRSB_all*-trans*,la1_) state formation with its photo-isomerization cycle without deprotonated retinal Schiff base RSB_13*-cis*_ formation was overlooked in [6] since no excitation intensity dependent photocycle experiments were carried out. With the new information on the excitation intensity dependent photocycle and photoproduct formation behavior for the thermally stable CaRh we think that the Ret_1 photocycle dynamics of BeRh is similar to the photocycle dynamics of CaRh. In the photo-isomerization scheme of Figure 9b in [6] the back-relaxation from TS_0_ to Ret_1 (PRSB_all*-trans*_) is thought to involve a meta-stable state Ret_1_la1_ (PRSB_all*-trans*,la1_) with all*-trans*—13*-cis* photo-isomerization cycling without PRSB_13*-cis*_ (Ret_5) reversible deprotonation to RSB (no Ret_4 formation).

### 3.3. Comparision of Photocycle Behavior of CaRh from Catenaria anguillulae with Photocycle Behavior of Channelrhodopsin ChR2 from Chlamydomonas reinhardtii

Photocurrent response studies to light stimuli and time resolved spectroscopy on channelrhodopsin ChR2 revealed a coupled dark-adapted state (D) and light-adapted desensitized state (Des) two-photocycle model [37]. Initially dark-adapted (IDA) ChR2-C128T mutant recovered to two different dark-adapted states DAB and DAG after blue and green light exposure, respectively (DAB = blue-light dark-adapted state, DAG = green-light dark-adapted state, IDA = initially dark-adapted state). The photo-excitation of DAB and DAG led to two coupled photocycles [38]. Liquid and solid-state nuclear magnetic resonance spectroscopy and resonance Raman spectroscopy on ChR2 were carried out to understand the substantial reduction of photocurrents during illumination, a process named “light-adaptation” [39]. It was shown that longer light pulse excitation led to an apparent dark-adapted state with two isomer conformations: all*-trans*,15-*anti* (IDA initial dark adapted state, D480) and 13*-cis*,15-*syn* (light-induced dark-adapted state, D470’). Both isomers together were named apparent dark-adapted state (DA_app_). The photo-excitation of both apparent dark-adapted state isomers caused two distinct photocycles [39].

The coupled photocycle occurrence of ChR2 has strong resemblance to the coupled dark-adapted-state G_da_ and light-adapted state G_la1_ photocycle behavior of CaRh from *Catenaria anguillulae* studied in this paper.

## 4. Materials and Methods

### 4.1. Sample Preparation

CaRh was expressed and purified as described earlier [4]. Briefly, the Rh domain (1–396 aa) of the full-length CaRhGC was expressed in *Pichia pastoris*. All purification steps were performed in 50 mM HEPES/MOPS buffer pH 7.5, 100 mM NaCl, 0.1 mM PMSF at 4 °C. Fractions that contained the protein were pooled, concentrated (Amicon Ultra 100 kDa, Millipore) to yield 2.6 mg/mL and stored at −80 °C.

### 4.2. Spectroscopic Investigations

The CaRh protein in pH 7.3 HEPES/MOPS buffer (20 mM 4-(2-hydroxyethyl)-1-piperazineethanesulfonic acid (HEPES), 20 mM 3-(*N*-morpholino) propanesulfonic acid (MOPS), 100 mM NaCl, 0.05 vol.% n-dodecyl β-d-maltoside (DDM), 0.01 vol.% cholesteryl hemisuccinate (CHS)) was stored at −80 °C. The nonionic detergent DDM and the ionizable anionic detergent CHS were used for good rhodopsin solubility and good thermal stability [40]. For usage CaRh was taken out of the −80 °C refrigerator, thawed and stored in the dark at about 3.5 °C. Absorption, fluorescence, photocycle and photo-degradation measurements were carried out at room temperature. The CaRh solutions were studied in fused silica ultra-micro cells (inner cell size 1.5 × 3 × 5 mm^3^, from Hellma Analytics, Müllheim, Germany). For sample centrifugation an Eppendorf Centrifuge 5702 R was used.

Transmission measurements, *T*(*λ*), were carried out with a spectrophotometer (Cary 50 from Varian). Attenuation coefficient spectra were calculated by the relation α(λ)=−ln[T(λ)]/l where *l* is the sample length. The attenuation coefficient α is composed of absorption, α_a_, and scattering, α_s_, contributions according to $\alpha (\lambda )=\alpha_{a}(\lambda )+\alpha_{s}(\lambda )$. α_s_(*λ*) is approximated by the empirical relation [7] $\alpha_{s}(\lambda )=\alpha (\lambda_{0}){\left(\lambda_{0}/\lambda \right)}^{\gamma }$ where *λ*_0_ is selected in the transparency region and γ ≤ 4 is fitted to the experimental attenuation in the transparency region (γ = 4 for small particles in the Rayleigh scattering regime, and γ < 4 for larger particle size in the Mie scattering regime). Absorption coefficient spectra α_a_(*λ*) became available by subtracting the scattering contribution α_s_(*λ*) from the measured attenuation coefficient spectra α(*λ*).

For fluorescence spectroscopic measurements a spectrofluorimeter (Cary Eclipse from Varian) was used (cell length in excitation direction 0.15 cm, cell width in detection direction 3 mm). Fluorescence quantum distributions *E*_F_(*λ*) were determined from fluorescence emission spectrum measurements at fixed excitation wavelengths [16,41,42]. The dye rhodamine 6G in methanol (fluorescence quantum yield Φ_F,ref_ = 0.94 [43]) was used as reference standard for fluorescence quantum distribution calibration. The fluorescence quantum yield is given by $\Phi_{F}=\int_{em}E_{F}(\lambda )d\lambda$ where the integration runs over the fluorescence emission wavelength region. The fluorescence spectra were deprived from scattering contributions by separate spectra measurements using a Ludox CL-X colloidal silica—water solution with particle size of 21 nm diameter and appropriate scattering contribution subtraction.

For absorption spectroscopic photocycle investigations, CaRh samples were excited with light emitting diodes LED 590 nm, LED 530 nm, and LED 470 nm from Thorlabs (spectral distributions included in Figure 7). The sample cell in the Cary 50 spectrophotometer was irradiated with the LEDs transverse to the transmission detection path (exposed area 3 × 5 mm^2^, sample thickness along excitation path 1.5 mm, transmission detection path length 3 mm). The excitation power *P*_exc_ was measured with a power meter (model PD 300-UV-SH photodiode detector head with NOVA power monitor from Ophir). Photo-degradation studies were carried out by long-time sample exposure with LED 590 nm, LED 530 nm, and LED 470 nm.

The apparent protein melting temperature of CaRh was determined by stepwise sample heating up and then cooling down, whereby transmission spectra were measured and the rising light scattering with sample heating was analyzed [21]. The thermal protein stability at room temperature (20.5 °C) and refrigerator temperature (3.5 °C) was determined by storing CaRh samples at the selected temperatures in the dark and measuring transmission spectra at certain time intervals whereby the temporal light attenuation development was analyzed.

## 5. Conclusions

The rhodopsin domain CaRh of the rhodopsin-guanylyl cyclase CaRhGC from *Catenaria anguillulae* was studied by absorption and emission spectroscopic methods. Its photophysical behavior was compared with that of BeRh, the rhodopsin domain of the rhodopsin-guanylyl cyclase BeRhGC from the aquatic fungus *Blastocladiella emersonii*. Both rhodopsin-guanylyl cyclases belong to the class of emzymerhodopsins in microbial organisms. Both CaRhGC and BeRhGC found already application as tools in optogenetics [4,5,29,30].

CaRh in pH 7.3 HEPES/MOPS buffer has the advantage of high thermal stability compared to BeRh in pH 8.0 Tris buffer of low thermal stability. The low thermal stability of BeRh may be the reason of the presence of a mixture of retinal—protein conformations in fresh thawed samples and conformational changes within the time of some hours at room temperature which show up in the UV-Vis absorption spectral shape of fresh thawed samples and the spectral development with time. On the other side the CaRh absorption spectrum of a fresh thawed sample exhibits inhomogeneous broadened singlet ground-state to first, second, and third singlet excited-state excitations with aggregation dependent changes on a several day timescale. The inhomogeneous absorption line broadening indicates a variation of the retinal structural shape and a variation of the arrangement and charge distribution of the surrounding amino acid residues of the opsin protein.

The photocycle dynamics of BeRh and CaRh by PRSB_all*-trans*_ excitation were found to behave quite similar. Sample exposure of dark-adapted BeRh and CaRh in the S_0_–S_1_ absorption band region of PRSB_all*-trans*_ caused (i) all*-trans*—13*-cis* photo-isomerization to PRSB_13*-cis*_, counter ion repositioning to PRSB_13*-cis*,cirp_, proton release to RSB_13*-cis*_ and recovery to PRSB_all*-trans*_ by re-protonation and *cis-trans* isomerization (primary photocycle) and (ii) it involved all*-trans* back-isomerization with protein restructuring to light-adapted PRSB_all*-trans*,la1_ with recovery in the dark to PRSB_all*-trans*_. Continued light exposure caused PRSB_all*-trans*,la1_ all*-trans*—13*-cis* photo-isomerization to PRSB_13*-cis*,la1_, counter ion repositioning to PRSB_13*-cis*,cirp,la1_ and *cis-trans* back-isomerization to PRSB_all*-trans*,la1_ without the involvement of proton release and re-protonation (secondary photocycle). The prolonged photo-excitation caused some low-efficient photo-degradation of the protonated retinal Schiff base.

## Figures and Tables

**Table 1:** Spectroscopic parameters of emitting parts of fresh thawed CaRh in pH 7.3 HEPES/MOPS buffer.

Transition	I	II	III	IV	V
	PRSB	PRSB	PRSB	PRSB	Apoprotein
	S_0_–S_1_	S_0_–S_1_	S_0_–S_2_	S_0_–S_3_	S_0_–S_1_
*λ*_a_ (nm)	540	450	410	370	280
*n*_a_	1.3334	1.3370	1.3386	1.3414	1.353
*λ*_F,max_ (nm)	≈690	≈525	≈588	≈425	≈330
$\Delta {\tilde{\nu }}_{F}$(cm^−1^)	≈6000	≈3000		≈4300	≈5000
${\bar{\lambda }}_{F}$(nm)	706	541	530	488	343
*n*_F_	1.3308	1.3334	1.3338	1.3355	1.3438
Φ_F_	1.1 × 10^−5^	8.5 × 10^−5^	2.0 × 10^−4^	4.75 × 10^−4^	0.045
${\bar{\sigma }}_{a}$(cm^2^)	3.3 × 10^−17^	3.3 × 10^−17^	≈3.3 × 10^−18^	≈3.3 × 10^−18^	3.0 × 10^−18^
*τ*_rad_ (ns)	8.0	3.6	≈37	≈29	9.95
*τ*_F_ (ps)	0.088	0.305	≈7.4	≈13.7	448

Abbreviations: *λ*_a_: absorption wavelength position. *n*_a_: mean refractive index in absorption region. *λ*_F,max_: wavelength position of fluorescence emission peak. $\Delta {\tilde{\nu }}_{F}$: spectral half-width of fluorescence emission spectrum (FWHM). ${\bar{\lambda }}_{F}$: mean fluorescence wavelength. *n*_F_: mean refractive index in fluorescence emission region. Φ_F_: fluorescence quantum yield. ${\bar{\sigma }}_{a}$: absorption band cross-section strength. *τ*_rad_: radiative lifetime. *τ*_F_: fluorescence lifetime.

**Table 2:** Primary retinal photocycle dynamics of CaRh in pH 7.3 HEPES/MOPS buffer (G_da_ → I → K → L → M → G_da_). Photo-excitation with LED 590 nm.

Parameter	Value	Comments
*λ*_a,max_(G_da_) (nm)	541	*t*_exc_ = 0, Figure 7
∆*λ*_a_(G_da_) (nm)	98.7	*t*_exc_ = 0, Figure 7
*λ*_a,max_(M) (nm)	365	Figure 7 and Figure 10
Φ*_cis_*	0.46 ± 0.05	Figure 8a and Figure S5, Equations (S11, S12, S13a, S13b)
Φ*_trans_*	0.54 ± 0.05	Φ*_trans_* = 1 − Φ*_cis_*
$\tau_{G_{da}^{*}\to Fu}$(ps)	≈0.088	$\tau_{G_{da}^{*}\to Fu}$≈ *τ*_F_
$\tau_{Fu\to I\to K}$(s)	<0.0125	Figure S5 and Figure 9a
$\tau_{K\to L}$(s)	0.048 ± 0.005	Figure S5 and Figure 9a
$\tau_{L\to M}$(s)	0.123 ± 0.005	Figure S5 and Figure 9b
τ_rec,la2_ (s)	0.35 ± 0.01	Figure S5 and Figure 9c
ĸ_la2_	≈0.27	1-ĸ_la1_, Equation (S9)
*I*_sat_ (W cm^−2^)	≈0.0129	Figure S4a

Abbreviations: *λ*_a,max_(G_da_): wavelength position of maximum absorption of S_0_–S_1_ band of CaRh_da_. Δ*λ*_a_: spectral half-width (FWHM) of S_0_–S_1_ band of CaRh_da_. *λ*_a,max_(M): wavelength position of maximum absorption of S_0_–S_1_ band of CaRh_la2_. Φ*_cis_*: quantum yield all*-trans* to 13*-cis* isomerization in CaRh photo-isomerization process. Φ*_trans_*: quantum yield of *trans* back-isomerization in CaRh photo-isomerization process. $\tau_{G_{da}^{*}\to Fu}$: time constant of excited-state relaxation from locally excited state LE to funnel state Fu of twisted internal conversion (conical intersection). *τ*_F__→I→K_: time constant of relaxation from funnel Fu via S_0_ transition state intermediate I to K (PRSB_13*-cis*_). *τ*_K→L_: time constant of relaxation of K (PRSB_13*-cis*_) to L (PRSB_13*-cis*,cirp_) by counter ion repositioning. *τ*_L→M_: time constant of relaxation of L (PRSB_13*-cis*,cirp_) to M (RSB_13*-cis*_) by proton release. *τ*_rec,la2_: recovery time constant of CaRh_la2_ (M) to CaRh_da_ (G_da_) by re-protonation and 13*-cis–*all*-trans* isomerization. ĸ_la2_: limiting fraction of CaRh_la2_ formation. *I*_sat_: excitation saturation intensity of CaRh_la2_ formation.

**Table 3:** Secondary photo-isomerization and protein restructuring photocycle of CaRh in pH 7.3 HEPES/MOPS buffer (G_la1_ → I → K → L → G_la1_ → G_da_). Photo-excitation with LED 590 nm.

Parameter	Value	Comments
*λ*_a,max_(G_la1_) (nm)	527	Figure 7
$\delta \lambda_{a,G_{da},G_{la1}}$(nm)	14	*λ*_a,max_(G_da_)–*λ*_a,max_(G_la1_)
$\delta \Delta \lambda_{a,G_{la1,}G_{da}}$(nm)	13.7	∆*λ*_a_(G_la1_)–∆*λ*_a_(G_da_)
ĸ_la1_	≈0.73	Figure 7b and Equation (S9)
*τ*_rec,la1_ (s)	0.8 ± 0.06	Figure S5, S6 and Figure 9a–e
*τ*_rec,*cis-trans*_ (s)	<<0.8	Figures S5 and S6
*I*_sat_ (W cm^−2^)	≈0.00595	Figure S4b
Φ_d_(w_exc_ = 3 J cm^−2^)	≈2.6×10^−4^	Figure 12

Abbreviations: *λ*_a,max_(G_la1_): wavelength position of maximum absorption of S_0_–S_1_ band of CaRh_la1_. $\delta \lambda_{a,G_{da},G_{la1}}$: wavelength shift of absorption maximum between CaRh_da_ and CaRh_la1_. $\delta \Delta \lambda_{a,G_{la1,}G_{da}}$: spectral broadening of first absorption band of CaRh_la1_ compared to CaRh_da_. ĸ_la1_: limiting fraction of CaRh_la1_ formation. *τ*_rec,la1_: recovery time constant of CaRh_la1_ (G_la1_) to CaRh_da_ (G_da_) by protein back-structuring. *τ*_rec,*cis-trans*_: recovery time constant of PRSB_13*-cis*,cirp,la1_ (L_la1_) to PRSB_all*-trans*,la1_ (G_la1_) by 13*-cis—*all*-trans* back-isomerization. *I*_sat_: excitation saturation intensity of CaRh_la1_ formation. Φ_d_: quantum yield of photo-degradation.

## Supplementary Materials

Supplementary materials can be found at www.mdpi.com/1422-0067/18/10/2099/s1.

## Abbreviations

BeRh	Rhodopsin domain of the rhodopsin-guanylyl cyclase from *Blastocladiella emersonii*
BeRhGC	Rhodopsin-guanylyl cyclase from *Blastocladiella emersonii*
cAMP	Cyclic adenosine monophosphate
CaRh	Rhodopsin domain of the rhodopsin-guanylyl cyclase from *Catenaria anguillulae*
CaRhGC	Rodopsin-guanylyl cyclase from *Catenaria anguillulae*
ChR2	Channelrhodopsin-2
cGMP	Cyclic guanosine monophosphate
CHS	Cholesteryl hemisuccinate
DDM	n-dodecyl β-d-maltoside
FWHM	Full width at half maximum
Fu	Funnel state
G	Ground-state
GTP	Guanosine triphosphate
HEPES	4-(2-Hydroxyethyl)-1-piperazineethanesulfonic acid
I	Intermediate state at TS_0_ transition state position
IC	Internal conversion
K	K-state in photocycle (PRSB_13*-cis*_)
L	L-state in photocycle (PRSB_13*-cis*,cirp_)
LE	Locally excited state
LED	Light emitting diode
M	M-state in photocycle (RSB_13*-cis*_)
MOPS	3-(*N*-morpholino) propanesulfonic acid
Prod	Photoproduct
PRSB	Protonated rerinal Schiff base
Ret_xxx_	Retinal photoproduct with absorption maximum at wavelength xxx nm
Rh-xxx	Rhodopsin isomer with absorption maximum at wavelength xxx nm
RSB	Retinal Schiff base
Trp	Tryptophan
TS_0_	Transition state on S_0_ ground-state potential energy surface
Tyr	Tyrosine

## Indices

cirp	Counter ion repositioned
da	Dark-adapted
F	Fluorescence
la	Light-adapted
la1	Light-adapted protonated retinal Schiff base ground-state
la2	Light-adapted deprotonated retinal Schiff base
Pr	Probe
Prod	Photoproduct

## Symbols

Symbol	Name	Unit
*E*_F_	Fluorescence quantum distribution	nm^−1^
*g*_LED_	Normalized spectral distribution of excitation LED	
*I*_exc_	Excitation light intensity	W cm^−2^
*I*_sat_	Excitation saturation intensity	W cm^−2^
*l*	Length	cm
*l*_exc_	Sample length in excitation direction	cm
*n*	Refractive index	
*N*	Number density	cm^−3^
*P*_exc_	Light excitation power	W
rpm	Rotations per minute	min^−1^
*t*	Time	s, min, or h
*T*	Transmission	
*t*_m_	Protein melting time	h or d
*t*_exc_	Time of sample excitation	s
*t*_res_	Time resolution	s
w*_exc_*	Excitation energy density	J cm^−2^
α	Attenuation coefficient	cm^−1^
α_a_	Absorption coefficient	cm^−1^
α_s_	Scattering coefficient	cm^−1^
*γ*	Scattering exponential factor	
*ϑ*	Temperature	°C
*ϑ*_m_	Apparent melting temperature	°C
ĸ_la1_	Limiting fraction of excited CaRh_da_* converted to CaRh_la1_ at high excitation intensity	
ĸ_la2_	Limiting fraction of excited CaRh_da_* converted to CaRh_la2_ at high excitation intensity	
*λ*	Wavelength	nm
*λ*_F,exc_	Fluorescence excitation wavelength	nm
*λ*_F,max_	Wavelength position of maximum fluorescence emission	nm
${\bar{\lambda }}_{F}$	Mean fluorescence wavelength	nm
ν	Frequency	Hz
$\tilde{\nu }$	Wavenumber	cm^−1^
$\delta {\tilde{\nu }}_{St}$	Fluorescence Stokes shift	cm^−1^
$\Delta {\tilde{\nu }}_{F}$	Fluorescence spectral half-width (FWHM)	cm^−1^
$\Delta {\tilde{\nu }}_{S_{0}-S_{1}}$	Absorption spectral half-width (FWHM) of S_0_–S_1_ transition	cm^−1^
σ_a_	Absorption cross-section	cm^2^
${\bar{\sigma }}_{a}$	Absorption band cross-section strength	cm^2^
*τ*_F_	Fluorescence lifetime	ps
*τ*_rad_	Radiative lifetime	ns
*τ*_rec_	Recovery time constant	s
Φ*_cis_*	Quantum yield of *trans-cis* isomerization	
Φ_d_	Quantum yield of photo-degradation	
Φ_F_	Fluorescence quantum yield	
Φ*_trans_*	Quantum yield of *trans* back isomerization	
∆*n*_ph,abs_	Increment of absorbed excitation photons	cm^−2^
*N*	Increment of length-integrated number density	cm^−2^

## References

[1] Gupta H.C., Singh K.P. (2002). Effect of sodium chloride on biology of *Catenaria anguillulae*. Mycobiology.

[2] Singh K.P., Vaish S.S., Kumar N., Singh K.D., Kumari M. (2012). *Catenaria anguillulae* as an efficient biological control agent of *Anguina tritici* in vitro. Biol. Control.

[3] Avelar G.M., Schumacher R.I., Zaini P.A., Leonard G., Richards T.A., Gomes S.L. (2014). A rhodopsin-guanylyl cyclase gene fusion functions in visual perception in a fungus. Curr. Biol..

[4] Gao S., Nagpal J., Schneider M.W., Kozjak-Pavlovic V., Nagel G., Gottschalk A. (2015). Optogenetic manipulation of cGMP in cells and animals by the tightly light-regulated guanylyl-cyclase opsin CyclOp. Nat. Commun..

[5] Scheib U., Broser M., Gao S., Constantin O., Stehfest K., Gee C.E., Oertner T.G., Nagel G., Hegemann P. (2017). Rhodopsin-cyclases for light-induced cGMP & cAMP production.

[6] Penzkofer A., Scheib U., Hegemann P., Stehfest K. (2016). Absorption and emission spectroscopic investigation of thermal dynamics and photo-dynamics of the rhodopsin domain of the rhodopsin-guanylyl cyclase from the aquatic fungus *Blastocladiella emersonii*. BAOJ Phys..

[7] Penzkofer A., Shirdel J., Zirak P., Breitkreuz H., Wolf E. (2007). Protein aggregation studied by forward light scattering and light transmission analysis. Chem. Phys..

[8] Ernst O.P., Lodowski D.T., Elstner M., Hegemann P., Brown L.S., Kandori H. (2014). Microbial and animal rhodopsins: Structures, functions, and molecular mechanisms. Chem. Rev..

[9] Penzkofer A., Luck M., Mathes T., Hegemann P. (2014). Bistable retinal Schiff base photodynamics of histidine kinase rhodopsin HKR1 from *Chlamydomonas reinhardtii*. Photochem. Photobiol..

[10] Strickler S.J., Berg R.A. (1962). Relationship between absorption intensity and fluorescence lifetime of molecules. J. Chem. Phys..

[11] Birks J.B., Dyson D.J. (1963). The relations between the fluorescence and absorption properties of organic molecules. Proc. Roy. Soc. London Ser. A.

[12] Deshpande A.V., Beidoun A., Penzkofer A., Wagenblast G. (1990). Absorption and emission spectroscopic investigation of cyanovinyldiethylaniline dye vapors. Chem. Phys..

[13] Lindsey J. PhotochemCAD spectra by category. http://omlc.ogi.edu/spectra/PhotochemCAD/html/.

[14] Turro N.J., Ramamurthy V., Scaiano J.C. (2009). Principles of Molecular Photochemistry. An introduction.

[15] Grabowski Z.R., Rotkiewicz K., Rettig W. (2003). Structural changes accompanying intramolecular electron transfer: Focus on twisted intramolecular charge-transfer states and structures. Chem. Rev..

[16] Förster T.H. (1951). Fluoreszenz Organischer Verbindungen.

[17] Valeur B. (2002). Molecular Fluorescence; Principles and Applications.

[18] Chen R.R. (1967). Fluorescence quantum yields of tryptophan and tyrosine. Anal. Lett..

[19] Eisinger J., Navon G. (1969). Fluorescence quenching and isotope effect in tryptophan. J. Chem. Phys..

[20] Kirby E.P., Steiner R.F. (1970). The influence of solvent and temperature upon the fluorescence of indole derivatives. J. Phys. Chem..

[21] Penzkofer A., Stierl M., Hegemann P., Kateriya S. (2011). Thermal protein unfolding in photo-activated adenylate cyclase nano-clusters from the amoeboflagellate *Naegleria gruberi* NEG-M strain. J. Photochem. Photobiol. A Chem..

[22] Voet D., Voet J.G. (2004). Biochemistry.

[23] Becker R.S., Freedman K. (1985). A comprehensive investigation of the mechanism and photophysics of isomerization of a protonated and unprotonated Schiff base of 11*-cis*-retinal. J. Am. Chem. Soc..

[24] Weigand R., Rotermund F., Penzkofer A. (1997). Aggregation dependent absorption reduction of indocyanine green. J. Phys. Chem. A.

[25] Fu P.P., Xia Q., Yin J.J., Cherng S.H., Yan J., Mei J., Chen T., Boudreau M.D., Howard P.C., Wamer W.G. (2007). Photodecomposition of vitamin A and photobiological implications to the skin. Photochem. Photobiol..

[26] Kateriya S., Nagel G., Bamberg E., Hegemann P. (2004). “Vision” in single-celled algae. News Physiol. Sci..

[27] Hegemann P. (2008). Algal sensory photoreceptors. Annu. Rev. Plant Biol..

[28] Luck M., Mathes T., Bruun S., Fudim R., Hagedorn R., Nguyen T.M.T., Kateriya S., Kennis J.T.M., Hildebrandt P., Hegemann P. (2012). A photochromic histidine kinase rhodopsin (HKR1) that is bimodally switched by ultraviolet and blue light. J. Biol. Chem..

[29] Scheib U., Stehfest K., Gee C.E., Körschen H.G., Fudim R., Oertner T.G., Hegemann P. (2015). The rhodopsin-guanylyl cyclase of the aquatic fungus *Blastocladiella emersonii* enables fast optical control of cGMP signaling. Sciencesignaling.

[30] Trieu M.M., Devine E.L., Lamarche L.B., Ammerman A.E., Greco J.A., Birge R.R., Theobald D.L., Oprian D.D. (2017). Expression, purification, and spectral tuning of RhoGC, a retenylidene/guanylyl cyclase fusion protein and optogenetics tool from the aquatic fungus *Blastocladiella emersonii*. J. Biol. Chem..

[31] Penzkofer A., Blau W. (1983). Theoretical analysis of S_1_-state lifetime measurements of dyes with picoseconds laser pulses. Opt. Quant. Electron..

[32] Penzkofer A. (1988). Solid state lasers. Prog. Quant. Electron..

[33] Longstaff C., Calhoon R.D., Rando R.R. (1986). Deprotonation of the Schiff base of rhodopsin is obligate in the activation of the G protein. Proc. Natl. Acad. Sci. USA.

[34] Lanyi J.K., Schobert B. (2002). Crystallographic structure of the retinal and the protein after deprotonation of the Schiff base: The switch in the bacteriorhodopsin photocycle. J. Mol. Biol..

[35] Kubli-Garfias C., Salazar-Salinas K., Perez-Angel E.C., Seminario J.M. (2011). Light activation of the isomerization and deprotonation of the protonated Schiff base retinal. J. Mol. Model..

[36] Lanyi J.K. (2004). Bacteriorhodopsin. Annu. Rev. Physiol..

[37] Stehfest K., Hegemann P. (2010). Evolution of the channelrhodopsin photocycle model. ChemPhysChem.

[38] Ritter E., Piwowarski P., Hegemann P., Bartl F.J. (2013). Light-dark adaptation of channelrhodopsin C128T mutant. J. Biol. Chem..

[39] Bruun S., Stoeppler D., Keidel A., Kuhlmann U., Luck M., Diehl A., Geiger M.A., Woodmansee D., Trauner D., Hegemann P. (2015). Light-dark adaptation of channelrhodopsin involves photoconversion between the all*-trans* and 13*-cis* retinal isomers. Biochemistry.

[40] De Grip W.J. (1982). Thermal stability of rhodopsin and opsin in some novel detergents. Methods Enzymol..

[41] Holzer W., Pichlmaier M., Penzkofer A., Bradley D.D.C., Blau W.J. (1999). Fluorescence spectroscopic behavior of neat and blended conjugated polymer thin films. Chem. Phys..

[42] Penzkofer A. (2012). Photoluminescence behavior of riboflavin and lumiflavin in liquid solutions and solid films. Chem. Phys..

[43] Sens R. (1984). Strahlungslose Desaktivierung in Xanthen-, Oxazin- und Carbazinfarbstoffen. Ph.D. Thesis.

