# Supplementary material for: Absorption and Emission Spectroscopic Investigation of Thermal Dynamics and Photo-Dynamics of the Rhodopsin Domain of the Rhodopsin-Guanylyl Cyclase from the Nematophagous Fungus Catenaria anguillulae
Source: Int J Mol Sci. 2017 Oct 5;18(10):2099. doi: 10.3390/ijms18102099 (PMC5666781; doi:10.3390/ijms18102099)
Supplement: Supplementary file 1 [file ijms-18-02099-s001.pdf]

International Journal of *Molecular Sciences*

MDPI

Supplementary Materials: Absorption and Emission Spectroscopic Investigation of Thermal Dynamics and Photo-Dynamics of the Rhodopsin Domain of the Rhodopsin-Guanylyl Cyclase from the Nematophagous Fungus *Catenaria anguillulae*

Alfons Penzkofer, Ulrike Scheib, Katja Stehfest and Peter Hegemann

S1. Amino Acid Sequence

The amino acid sequence of the here investigated recombinant synthesized rhodopsin CaRh is displayed in Figure S1. It was added MS at the N-terminus and ENLYFQGVDHHHHHH at the C-terminus to the Rh domain of the full-length CaRhGC protein. Its apoprotein molar mass is M_{pr} = 45484.94 g mol^{-1}. It contains 14 Tyr, 12 Trp, and 26 Phe residues.

```
        10         20         30         40         50         60
MSMKDKDNNL RGACSGCSCP EYCYSPTSTL CDDCKCSVTK HPIVEQPLTR NGSFRSSGAS
        70         80         90        100        110        120
LLPSPSQPNI KVTGSSTASS NANMRNRQNN SLSVSNVRST SSASSSNVSS PANSRPGSPS
       130        140        150        160        170        180
KQSALQQYQT NIADMWSWDM MLSTPSLKFL TGQFIMWAIL TVAGAFYALF IQERQAYNRG
       190        200        210        220        230        240
WADIWYGYGA FGFGIGIAFS YMGFAGARNP EKKALSLCLL GVNIIAFSSY ILIMLRLTPT
       250        260        270        280        290        300
IEGTLSNPVE PARYLEWIAT CPVLILLISE ITQADHNAWG VVFSDYALVV CGFFGAVLPP
       310        320        330        340        350        360
YPWGNLFNIL SCAFFSFVVY SLWRSFTGAI NGETPCNIEV NGLRWTRFST VTTWTLFPLS
       370        380        390        400        410
WFAFTSGMLS FTMTEASFTM IDIGAKVFLT LVLVNSTVEN LYFQGVDHHH HHH
```

Figure S1. Amino acid sequence of CaRh

S2. Absorption Cross-Section Determination

The absorption cross-section spectrum shape $\sigma_{a,CaRh}(\lambda)$ of CaRh (solid curve in top part of Figure S2) is equal to the absorption coefficient shape $\alpha_a(\lambda)$ of Figure 1. The absolute absorption cross-section spectrum $\sigma_{a,CaRh}(\lambda)$ of CaRh is determined by setting the absorption cross-section of CaRh at λ = 270 nm equal to the apoprotein Trp, Tyr, and Phe absorption cross-section contribution $\sigma_{26F+14Y+12W}$(270 nm) and some estimated retinal absorption cross-section contribution [S1]. The involved absorption cross-section spectra of Phe, Tyr, and Trp were taken from [13]. The apoprotein

absorption cross-section spectrum $\sigma_{26F+14Y+12W}$ of CaRh is shown by the dashed curve in the top part of Figure S2. It was calculated as the sum of the absorption cross-section spectra of 26 Phe, 14 Tyr, and 12 Trp residues present in one apoprotein. The CaRh molecule number density was determined by

$$N_{CaRh} = \frac{\alpha_{a,apo}(270\ nm)}{\sigma_{26\ F+14Y+12W}(270\ nm)} = \frac{\alpha_{a,CaRh}(270\ nm) - \alpha_{a,retinal}(270\ nm)}{\sigma_{26\ F+14Y+12W}(270\ nm)}, \quad (S1)$$

and the absorption cross-section spectrum of CaRh was set to

$$\sigma_{a,CaRh}(\lambda) = \frac{\alpha_{a,CaRh}(\lambda)}{N_{CaRh}}. \quad (S2)$$

The values used in the calculations were $\alpha_{a,CaRh}(270\ nm) = 17.22\ cm^{-1}$, $\alpha_{a,retinal}(270\ nm) = 1.8\ cm^{-1}$, and $\sigma_{26F+14Y+12W}(270\ nm) = 3.08\times10^{-16}\ cm^2$ giving $N_{CaRh} = 5.01\times10^{16}\ cm^{-3}$.

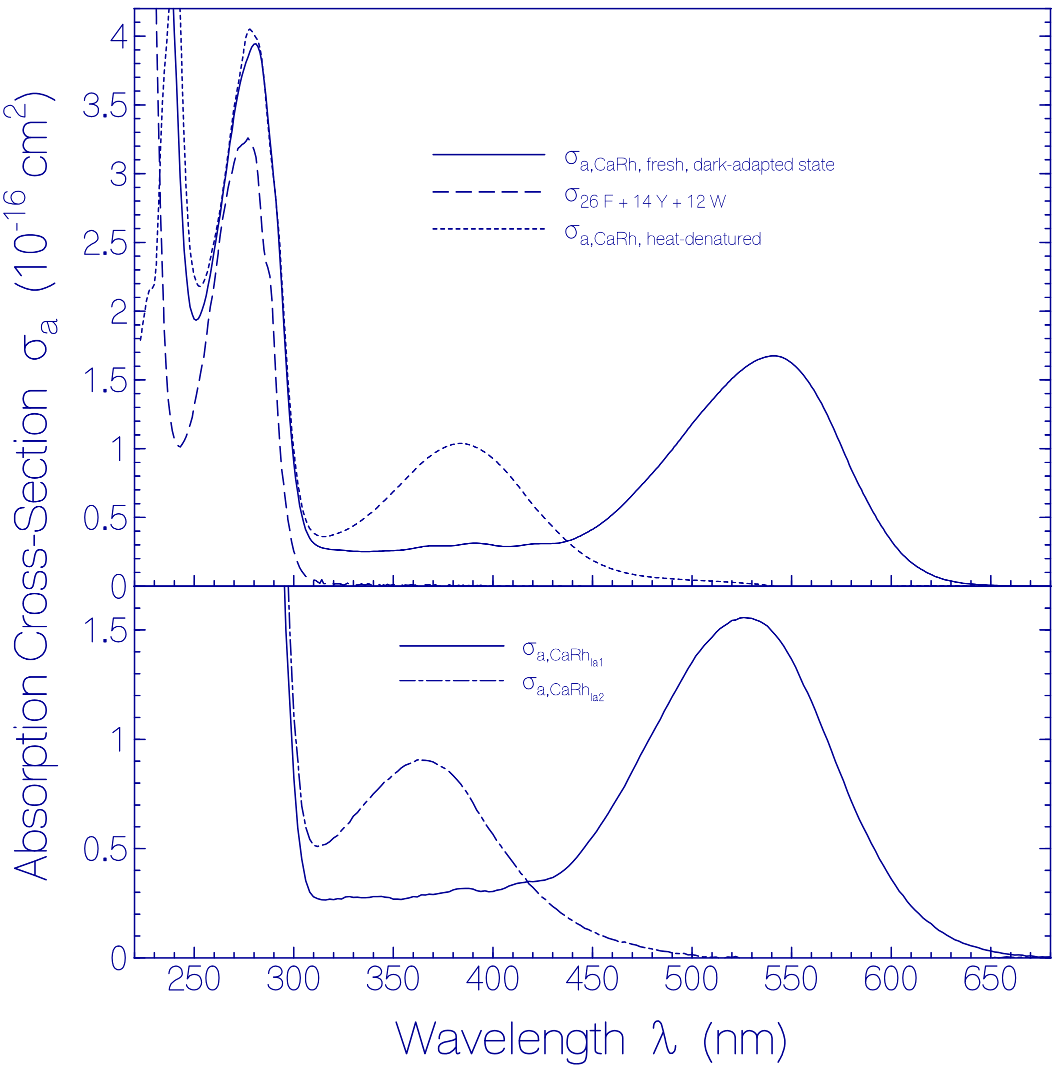

Figure S2. Absorption cross-section spectra. Curves are identified by the legends.

The absorption cross-section spectrum $\sigma_{a,CaRh,fresh,\ dark-adapted\ state}(\lambda > 310\ nm)$ is the absorption cross-section spectrum of PRSB in CaRh for $\lambda > 310$ nm. The determination of $\sigma_{a,CaRh,\ heat-denatured}$ is described in section 2.2.1 of the main text. The main band with absorption maximum at 384 nm is

the S_0-S_1 absorption cross-section band of RSB in heat-denatured CaRh. The absorption cross-section spectrum $\sigma_{a,CaRh_{la1}}(\lambda)$ is the absorption cross-section spectrum of CaRh in its light-adapted ground-state la1 (G_{la1}). Its determination is described in section S7. $\sigma_{a,CaRh_{la1}}(\lambda > 310\ nm)$ is caused by the excitation of $PRSB_{all\text{-}trans,la1}$. The absorption cross-section spectrum $\sigma_{a,CaRh2}(\lambda)$ is the absorption cross-section spectrum of CaRh in its light-adapted state la2 ($CaRh_{la2}$). Its determination is described in section S7. The main band with absorption maximum at 365 nm is the S_0-S_1 absorption cross-section band of $RSB_{13\text{-}cis}$ in $CaRh_{la2}$.

S3. Nano-Cluster Size of a Fresh Centrifuged CaRh Sample

The nano-cluster size of fresh CaRh is determined analogous to the description in [6] and [S2]. The scattering cross-section σ_s is obtained from the scattering coefficient α_s by $\sigma_s = \alpha_s/N_{CaRh}$. For the sample used in Figure 1, at λ = 632.8 nm it is $\alpha_s(\lambda) = \alpha_s(\lambda_0)(\lambda_0/\lambda)^\gamma$ = 0.0702 cm^{-1} (λ_0 = 800 nm, $\alpha_s(\lambda_0)$ = 0.046 cm^{-1}, γ = 1.8) and $\sigma_s(\lambda) = 1.40\times10^{-18}$ cm^2 ($N_{CaRh} = 5.01\times10^{16}$ cm^{-3}).

The scattering cross-section σ_s is theoretically given by [7]

$$\sigma_s = M_{sca}\sigma_{R,m} = \beta_m\widetilde{M}\sigma_{R,m} \tag{S3}$$

where $M_{sca} = \beta_m\widetilde{M}$ is the aggregation scattering enhancement factor, β_m is the degree of aggregation (average number of protein molecules per cluster particle), $\widetilde{M}$ is the total Mie scattering function ($\widetilde{M} \le 1$ decreasing with increasing aggregate size [7]), and $\sigma_{R,m}$ is the monomer Rayleigh scattering cross-section. The monomer Rayleigh scattering cross-section is given by [7]

$$\sigma_{R,m}(\lambda) = \frac{8\pi}{3}\frac{4\pi^2 n_s^4}{\lambda^4}V_m^2\left(\frac{n_{pr}^2 - n_s^2}{n_{pr}^2 + 2n_s^2}\right)^2 = \frac{8\pi}{3}\frac{4\pi^2 n_s^4}{\lambda^4}\left(\frac{M_{pr}}{N_A\rho_{pr}}\right)^2\left(\frac{n_{pr}^2 - n_s^2}{n_{pr}^2 + 2n_s^2}\right)^2. \tag{S4}$$

Thereby n_s is the refractive index of the solvent (water buffer) at wavelength λ, n_{pr} is the refractive index of the protein at wavelength λ, $V_m = M_{pr}/(N_A\rho_{pr})$ is the volume of one protein molecule, M_{pr} is the molar mass of the protein monomer (M_{pr} = 45484.94 g mol^{-1} for CaRh apoprotein), N_A = 6.022142×10^{23} mol^{-1} is the Avogadro constant, and ρ_{pr} is the mass density of the protein (typical value for proteins is $\rho_{pr} \approx 1.412$ g cm^{-3} [S3]). These numbers give a protein monomer volume of $V_m \approx$ 53.49 nm^3 and a protein monomer radius of $a_m = [3V_m/(4\pi)]^{1/3} \approx 2.34$ nm. At λ = 632.8 nm there is n_s = 1.332 and $n_{pr} \approx 1.589$ [S4] giving $\sigma_{R,m}$(632.8 nm) = 2.838×10^{-21} cm^2. Insertion into Equation (S3) gives $M_{sca} = \beta_m\widetilde{M} = \sigma_s/\sigma_{R,m} \approx 494$. The small value of γ = 1.8 indicates a small $\widetilde{M}$ and a large cluster volume $V_{ag} = \beta_m V_m/\kappa_{f,m}$ with small volume fill factor $\kappa_{f,m}$ [7].

S4. Fluorescence Quantum Distribution of Heat-Denatured CaRh

The fluorescence quantum distribution of the heat-denatured CaRh sample of Figure 4 is shown in Figure S3. The corresponding attenuation coefficient spectrum is shown by the thick solid curve in Figure 4a (4 °C, end). Fluorescence excitation occurred at $\lambda_{F,exc}$ = 360 nm.

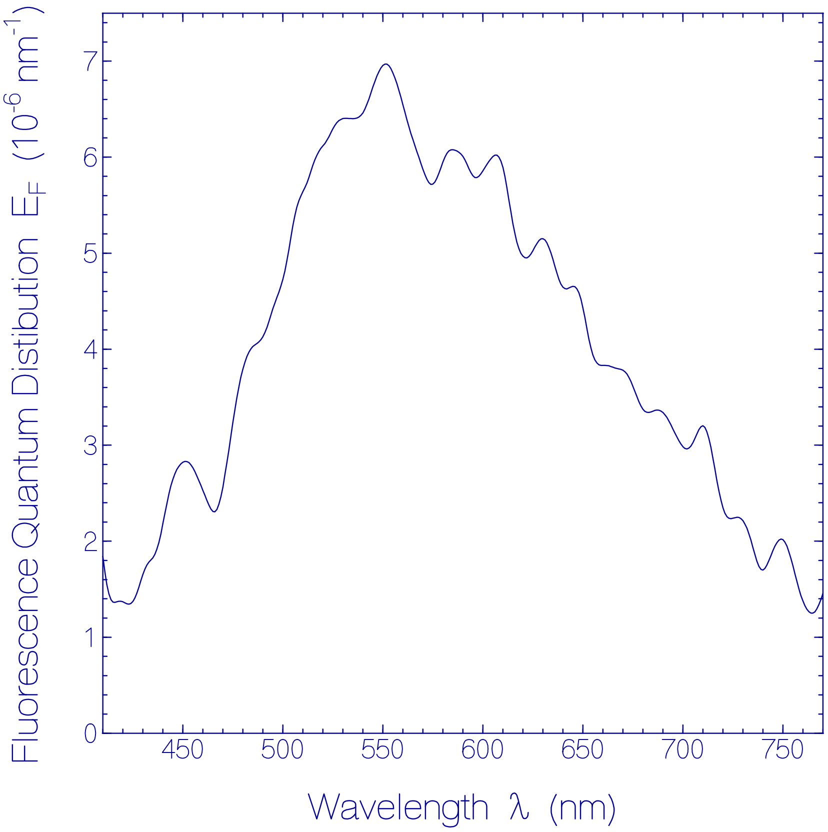

Figure S3. Fluorescence quantum distribution of heat-denatured CaRh in pH 7.3 HEPES/MOPS buffer for fluorescence excitation wavelength $\lambda_{F,exc}$ = 360 nm (excitation of RSB, belongs to thick solid curve of attenuation coefficient spectrum shown in Figure 4a).

S5. Excitation intensity dependent steady-state attenuation coefficient changes

The attenuation coefficient change $\delta\alpha(\lambda_{pr}, \lambda_{exc}, I_{exc}) = \alpha_{extremum}(\lambda_{pr}, \lambda_{exc}, I_{exc}) - \alpha(\lambda_{pr}, I_{exc} = 0)$ versus excitation intensity for the excitation wavelengths λ_{exc} = 530 nm (LED 530 nm), 590 nm (LED 590 nm) and 470 nm (LED 470 nm) is shown in Figure S4a for λ_{pr} = 370 nm and in Figure S4b for λ_{pr} = 550 nm. The circles are experimental data. The curves are nonlinear regression fits to the experimental data using the relation [S5]

$$\delta\alpha(\lambda_{pr}, \lambda_{exc}, I_{exc}) = \delta\alpha_0(\lambda_{pr}) \frac{I_{exc} / I_{sat}(\lambda_{exc}, \lambda_{pr})}{1 + I_{exc} / I_{sat}(\lambda_{exc}, \lambda_{pr})}, \quad \text{(S5)}$$

with $\delta\alpha_0$ and $I_{sat}(\lambda_{exc}, \lambda_{pr})$ listed in the sub-figures. The saturation intensity is inverse proportional to absorption cross-section $\sigma_a(\lambda_{exc})$ and the absorption recovery time τ_{rec} [S5].

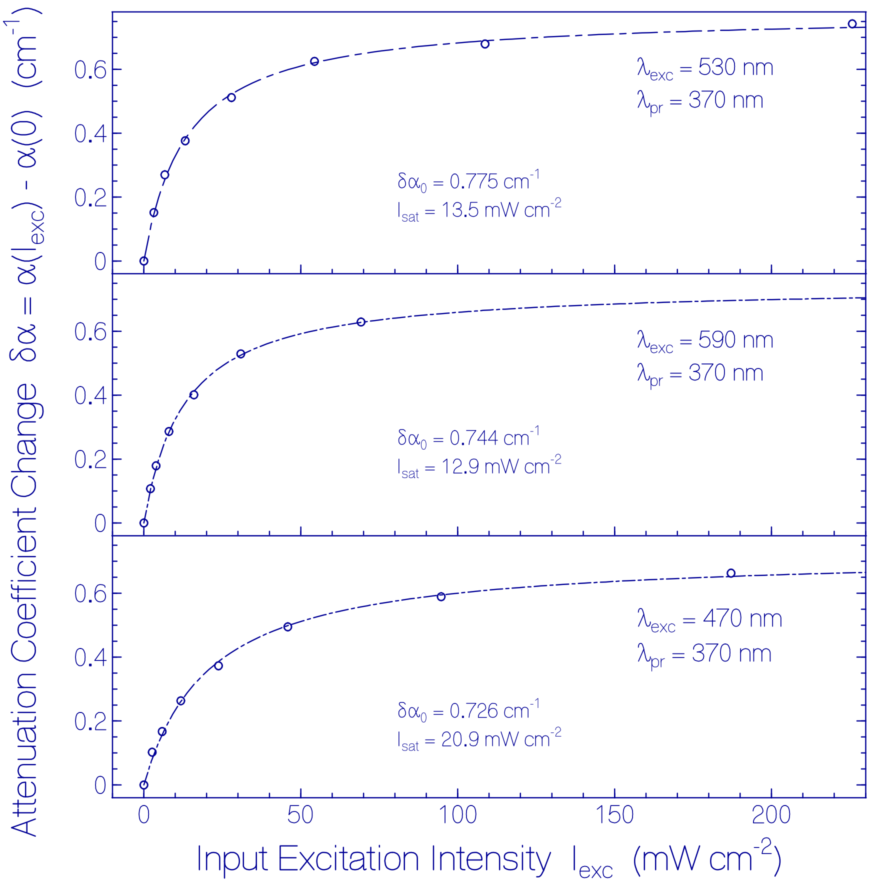
Attenuation Coefficient Change δα = α(I_exc) - α(0) (cm^-1)
λexc = 530 nm
λpr = 370 nm
δα0 = 0.775 cm-1
Isat = 13.5 mW cm-2
λexc = 590 nm
λpr = 370 nm
δα0 = 0.744 cm-1
Isat = 12.9 mW cm-2
λexc = 470 nm
λpr = 370 nm
δα0 = 0.726 cm-1
Isat = 20.9 mW cm-2
Input Excitation Intensity Iexc (mW cm-2)

(**a**)

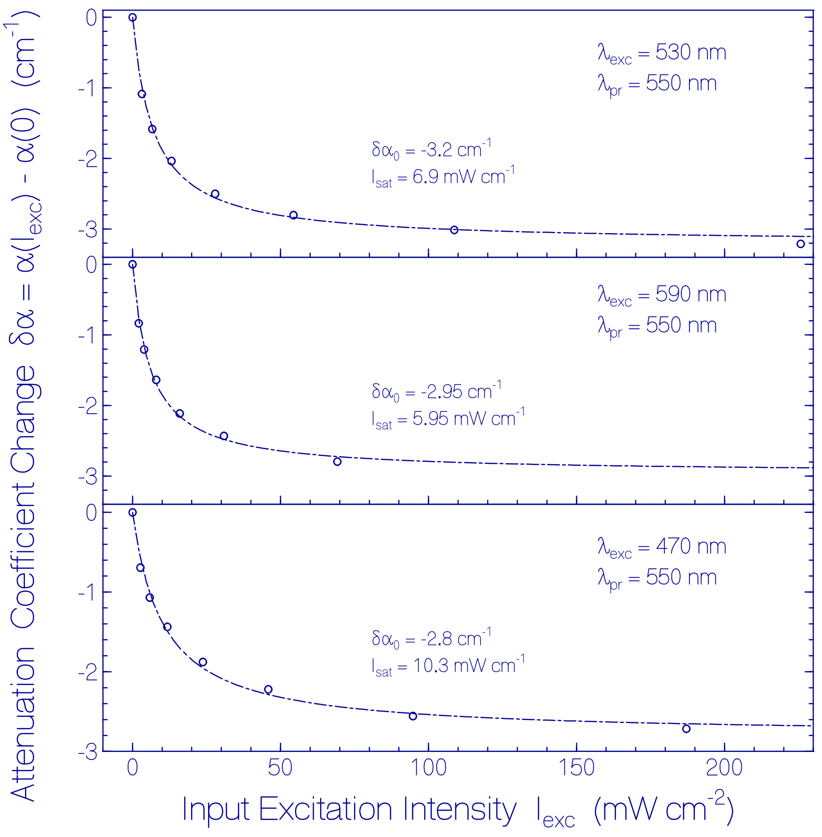
Attenuation Coefficient Change δα = α(I_exc) - α(0) (cm^-1)
λexc = 530 nm
λpr = 550 nm
δα0 = -3.2 cm-1
Isat = 6.9 mW cm-1
λexc = 590 nm
λpr = 550 nm
δα0 = -2.95 cm-1
Isat = 5.95 mW cm-1
λexc = 470 nm
λpr = 550 nm
δα0 = -2.8 cm-1
Isat = 10.3 mW cm-1
Input Excitation Intensity Iexc (mW cm-2)

(**b**)

Figure S4. Dependence of attenuation coefficient change $\delta\alpha(\lambda_{pr}, \lambda_{exc}, I_{exc}) = \alpha_{extremum}(\lambda_{pr}, \lambda_{exc}, I_{exc})$ - $\alpha(\lambda_{pr}, I_{exc} = 0)$ of CaRh in pH 7.3 HEPES/MOPS buffer (**a**) at λ_{pr} = 370 nm and (**b**) at λ_{pr} = 550 nm on excitation light intensity I_{exc} for λ_{exc} = 530 nm (top part), 590 nm (middle part), and 470 nm (bottom part). Circles are experimental data. The curves are nonlinear regression fits to the experimental data using the relation $\delta\alpha(\lambda_{pr}, I_{exc}) = \delta\alpha_0 (I_{exc}/I_{sat})/(1 + I_{exc}/I_{sat})$ with $\delta\alpha_0$ and I_{sat} listed in the sub-figures.

S6. Calculation of quantum yield of photo-degradation

The quantum yield of photodegradation ϕ_d is given by

$$\phi_d = \frac{\Delta N_{da}}{\Delta n_{ph,abs}}, \qquad \text{(S6)}$$

where ΔN_{da} is the increment of length-integrated number density of degraded dark-adapted CaRh (Rh-541) and $\Delta n_{ph,abs}$ is the increment of absorbed excitation photons by light-adapted CaRh (Rh-527).

ΔN_{da} is given by

$$\Delta N_{da} = \overline{N}_{da} l_{exc} \frac{\Delta\alpha_a(\lambda_{pr})}{\overline{\alpha}_a(\lambda_{pr})} = \frac{\overline{\alpha}_a(\lambda_{pr})}{\sigma_{a,da}(\lambda_{pr})} l_{exc} \frac{\Delta\alpha_a(\lambda_{pr})}{\overline{\alpha}_a(\lambda_{pr})} = l_{exc} \frac{\Delta\alpha_a(\lambda_{pr})}{\sigma_{a,da}(\lambda_{pr})}. \qquad \text{(S7)}$$

$\overline{N}_{da} = \overline{\alpha}_a(\lambda_{pr}) / \sigma_{a,da}(\lambda_{pr})$ is the average number density of CaRh in the dark-adapted state. $\overline{\alpha}_a(\lambda_{pr}) = [\alpha_{a,\, begin\ of\ exposure\ interval}(\lambda_{pr}) + \alpha_{a,\, recovered\ after\, end\ of\ exposure\ interval}(\lambda_{pr})]/2$ is the average absorption coefficient of dark-adapted CaRh at λ_{pr} (here used $\lambda_{pr} = \lambda_{da,max}$ = 541 nm). l_{exc} is the sample length in excitation direction, $\Delta\alpha_a(\lambda_{pr}) = \alpha_{a,\, begin\ of\ exposure\ interval}(\lambda_{pr}) - \alpha_{a,\, recovered\ after\, end\ of\ exposure\ interval}(\lambda_{pr})$ is the absorption coefficient change of dark-adapted CaRh at the probe wavelength λ_{pr} due to the photon absorption $\Delta n_{ph,abs}$ in the considered excitation interval of CaRh in the light-adapted state.

The increment of absorbed excitation photons $\Delta n_{ph.abs}$ in the considered time increments δt_{exc} is

$$\Delta n_{ph,abs} = \frac{I_{exc}\delta t_{exc}}{h\nu_{exc}} \left[1 - \exp\left(-\overline{\alpha}_{a,exc,la} l_{exc}\right)\right], \qquad \text{(S8a)}$$

where $\overline{\alpha}_{a,exc,la}$ is the absorption coefficient of the light-adapted sample (Rh-527 is absorbing) averaged over the spectral distribution of the excitation light source $g_{LED,i}$ and averaged over excitation time interval δt_{exc}, i.e.,

$$\overline{\alpha}_{a,exc,la} = \frac{\int \alpha_{a,\, at\ \delta t_{exc}/2}(\lambda) g_{LED,i}(\lambda) d\lambda}{\int g_{LED,i}(\lambda) d\lambda}. \qquad \text{(S8b)}$$

S7. Determination of photocycle parameters

The limiting fraction κ_{la1} of excited CaRh$_{da}$* converted to CaRh$_{la1}$ at high excitation intensity is obtained from the ratio of the absorption coefficient strength of the S_0-S_1 transition of CaRh$_{la1}$ at high excitation intensity (dashed curves in Figure 7 for t_{exc} = 3 s) to the initial absorption coefficient strength of the S_0-S_1 transition of CaRh$_{da}$ before excitation (solid curves in Figure 7). That is

$$\kappa_{la1} \approx \frac{\int_{S_0-S_1} \frac{\alpha_{a,CaRh_{la1}}(\lambda, I_{exc} \to \infty)}{\lambda} d\lambda}{\int_{S_0-S_1} \frac{\alpha_{a,CaRh_{da}}(\lambda, I_{exc} = 0)}{\lambda} d\lambda} . \quad \text{(S9)}$$

(S_0-S_1 upper wavelength position in the integration is set to $\lambda_{upper\ limit}$ = 430 nm). Thereby it is assumed that the absorption cross-section strengths of the S_0-S_1 transiton of CaRh$_{da}$ and CaRh$_{la1}$ are approximtely equal. The analysis gives $\kappa_{la1} \approx 0.73$ for λ_{exc} = 530 nm, 590 nm, and 470 nm. The limiting fraction κ_{la2} of excited CaRh$_{da}$* converted to CaRh$_{la2}$ is $\kappa_{la2} = 1 - \kappa_{la1} \approx 0.27$ for λ_{exc} = 530 nm, 590 nm, and 470 nm.

Considering the photocycle scheme of Figure 13a and the reaction coordinate scheme of Figure S5 the ratio of $\kappa_{la2}/\kappa_{la1}$ is given by

$$\frac{\kappa_{la2}}{\kappa_{la1}} = \frac{\phi_{cis}\tau_{rec,la2}}{\phi_{trans}\tau_{rec,la1}} = \frac{\phi_{cis}\tau_{rec,la2}}{(1-\phi_{cis})\tau_{rec,la1}} . \quad \text{(S10)}$$

The κ_{la2} and κ_{la1} values obtained from Equation (S9) give $\kappa_{la2}/\kappa_{la1} \approx 0.37$. Application of Equation (S10) gives $\kappa_{la2}/\kappa_{la1} = 0.37 \pm 0.13$ using $\phi_{cis} = 0.46 \pm 0.05$, $\tau_{rec,la2} = 0.35 \pm 0.01$ s and $\tau_{rec,la1} = 0.8 \pm 0.06$ s.

The absorption coefficient spectrum of α_a(λ, t_{exc} = 3 s, λ_{exc} = 530 nm, I_{exc} = 226 mW cm^{-2}) of Figure 7a is approximately separated in the absorption coefficient contributions $\alpha_{a,CaRh_{la1}}(\lambda)$ and $\alpha_{a,CaRh_{la2}}(\lambda)$ which are shown by the thick dashed and the thick dotted curves in Figure 7a (shape of $\alpha_{a,CaRh_{la2}}(\lambda)$ is taken from initial photo-degradation development of Figure 10). The absorption cross-section spectra of CaRh$_{la1}$ and CaRh$_{la2}$ are given by $\sigma_{a,CaRh_{la1}}(\lambda) = \alpha_{a,CaRh_{la1}}(\lambda)/N_{CaRh_{la1}}$ and $\sigma_{a,CaRh_{la2}}(\lambda) = \alpha_{a,CaRh_{la2}}(\lambda)/N_{CaRh_{la2}}$. The number density of CaRh$_{la1}$ is given by $N_{CaRh_{la1}} = N_{CaRh,0}\kappa_{la1}$ $\approx 3.66 \times 10^{16}$ cm^{-1}, and the number density of CaRh$_{la2}$ is given by $N_{CaRh_{la2}} = N_{CaRh,0}\kappa_{la2}$ $\approx 1.35 \times 10^{16}$ cm^{-3} ($N_{CaRh,0} = \alpha_a(541\,nm, t_{exc} = 0)/\sigma_{a,CaRh_{da}}(541\,nm)$ $\approx 5.01 \times 10^{16}$ cm^{-3} with α_a(541 nm, t_{exc} = 0) = 8.4 cm^{-1} of Figure 7a, $\sigma_{a,CaRh_{da}}(541\,nm)$ = 1.675×10^{-16} cm^2, see top part of Figure S2). The obtained approximate absorption cross-section spectra of CaRh$_{la1}$ and CaRh$_{la2}$ are shown in the bottom part of Figure S2.

The initial quantum yield of all-*trans* – 13-*cis* photo-isomerization ϕ_{cis} (Figure S5) of CaRh$_{da}$ is deduced from the initial light induced absorption change at λ_{pr} = 550 nm of middle part of Figure 8a for λ_{exc} = 590 nm and t_{exc} = 0.0125 s. ϕ_{cis} is approximately given by

$$\phi_{cis} = \frac{\Delta N_{da}}{\Delta n_{ph,abs}} , \quad \text{(S11)}$$

where ΔN_{da} is the increment of length-integrated number density of all-*trans* – 13-*cis* isomerized initially dark-adapted CaRh, and $\Delta n_{ph,abs}$ is the increment of absorbed excitation photons by initially dark-adapted CaRh (Rh-541).

ΔN_{da} is given by

$$\Delta N_{da} = N_{da} l_{exc} \frac{\Delta\alpha_a(\lambda_{pr})}{\alpha_a(\lambda_{pr})} = \frac{\alpha_a(\lambda_{pr})}{\sigma_{a,da}(\lambda_{pr})} l_{exc} \frac{\Delta\alpha_a(\lambda_{pr})}{\alpha_a(\lambda_{pr})} = l_{exc} \frac{\Delta\alpha_a(\lambda_{pr})}{\sigma_{a,da}(\lambda_{pr})} \quad \text{(S12)}$$

$N_{da} = \alpha_a(\lambda_{pr}) / \sigma_{a,da}(\lambda_{pr})$ is the number density of CaRh in the dark-adapted state. $\alpha_a(\lambda_{pr})$ is the absorption coefficient of dark-adapted CaRh at λ_{pr} (here used λ_{pr} = 550 nm). l_{exc} is the sample length in excitation direction (here l_{exc} = 0.15 cm), $\Delta\alpha_a(\lambda_{pr})$ is the absorption coefficient change of dark-adapted CaRh at the probe wavelength λ_{pr} due to the photon absorption $\Delta n_{ph,abs}$ within t_{exc} = 0.0125 s.

The increment of absorbed excitation photons $\Delta n_{ph.abs}$ in the considered time increment t_{exc} is

$$\Delta n_{ph,abs} = \frac{I_{exc} t_{exc}}{h\nu_{exc}} \left[1 - \exp\left(-\overline{\alpha}_{a,da}(\lambda_{exc}) l_{exc}\right)\right], \quad \text{(S13a)}$$

where $\overline{\alpha}_{a,da}(\lambda_{exc})$ is the absorption coefficient of the dark-adapted sample averaged over the spectral distribution of the excitation light source $g_{LED590nm}$, i.e.,

$$\overline{\alpha}_{a,da}(\lambda_{exc}) = \frac{\int \alpha_{a,da}(\lambda) g_{LED590nm}(\lambda) d\lambda}{\int g_{LED590nm}(\lambda) d\lambda}. \quad \text{(S13b)}$$

The obtained quantum yield is ϕ_{cis} = 0.46±0.05. The quantum yield of all-*trans* back-isomerization is $\phi_{trans} = 1 - \phi_{cis}$ = 0.54±0.05.

S8. Schematic Reaction Coordinate Diagrams for Primary and Secondary Photo-Isomerization Cycles of CaRh

A schematic reaction coordinate diagram for the primary photo-isomerization and deprotonation/re-protonation cycle together with the back-*trans* isomerization with protein restructuring of initially dark-adapted CaRh is shown in Figure S5. The reaction coordinate resembles the diheadral angle of the C13=C14 bond of retinal [8,9]. In Figure S6 a schematic reaction coordinate diagram for the secondary photo-isomerization cycle of light-adapted $CaRh_{la1}$ ($PRSB_{all\text{-}trans,la1}$) is depicted.

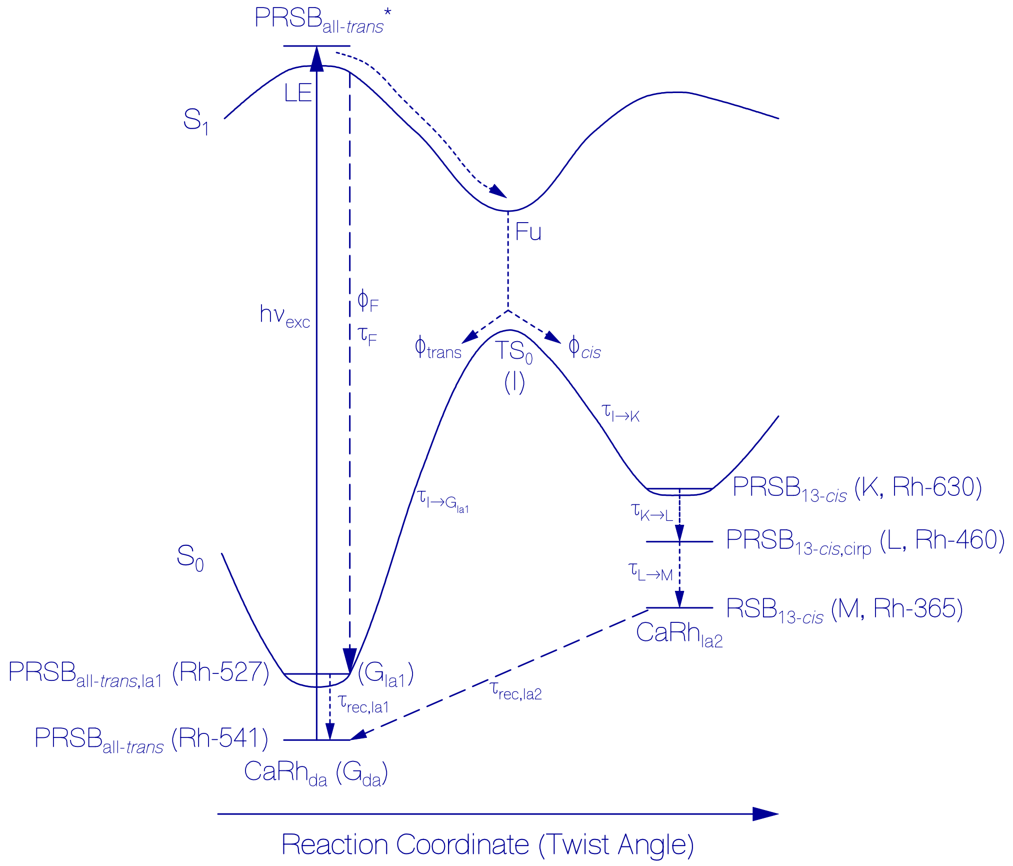

Figure S5. Schematic reaction coordinate diagram for primary photocycle of CaRh in pH 7.3 HEPES/MOPS buffer.

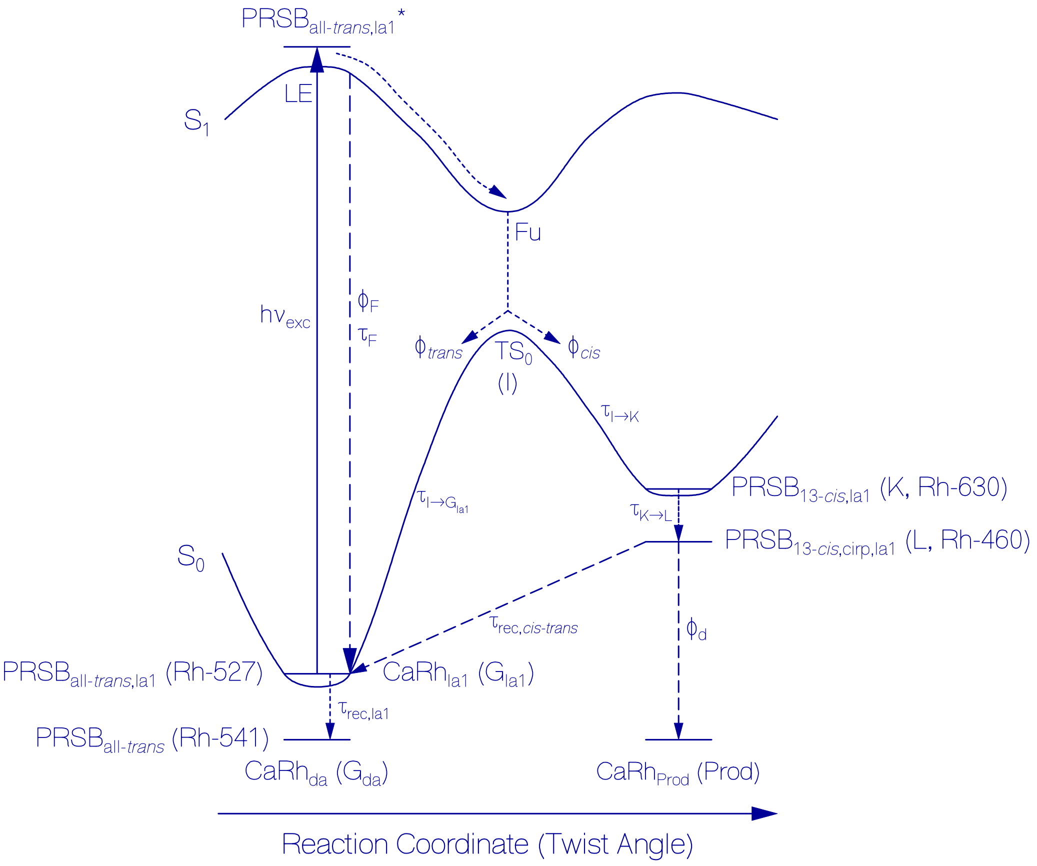

Figure S6. Schematic reaction coordinate diagram for secondary photocycle of CaRh in pH 7.3 HEPES/MOPS buffer.

References

S1. Honig, B.; Dinur, U.; Birge, R.R.; Ebrey, T.G. The isomer dependence of oscillator strengths in retinal and related molecules. Spectroscopic assignments. *J. Am. Chem. Soc.* **1980**, *102*, 488–494.

S2. Penzkofer, A.; Stierl, M.; Hegemann, P.; Kateriya, S. Absorption and fluorescence characteristics of photo-activated adenylate cyclase nano-clusters from the amoeboflagellate *Nagleria gruberi* NEG-M strain. *Chem. Phys.* **2012**, *392*, 46–54.

S3. Fischer, H.; Polikarpov, I.; Craievich, A. Average protein density is a molecular-weight-dependent function. *Protein Sci.* **2004**, *13*, 825–828.

S4. Barer, R.; Tkaczyk, S. Refractive index of concentrated protein solutions. *Nature* **1954**, *173*, 821–822.

S5. Penzkofer, A.; Stierl, M.; Mathes, T.; Hegemann, P. Absorption and emission spectroscopic characterization of photo-dymanics of photoactivated adenylyl cyclase mutant bPAC-Y7F of *Beggiatoa* sp. *J. Photochem. Photobiol. B Biol.* **2014**, *140*, 182–193.
